# Multimodal emotion recognition using hybrid deep feature fusion under speaker-independent evaluation

**DOI:** 10.1038/s41598-026-58836-w

**Published:** 2026-06-25

**Authors:** Elhossiny Ibrahim, Mohamed Ezzat Ghoraba, Ahmed Ezzat Ghoraba

**Affiliations:** 1 Department of Computer Science and Engineering, Faculty of Electronic Engineering, Menoufia University, Menouf, Egypt; 2 Department of Computer Engineering and Automatic Control, Faculty of Engineering, Tanta University, Tanta, Egypt; 3 Department of Artificial Intelligence, Faculty of Engineering, Tanta University, Tanta, Egypt

**Keywords:** Multimodal emotion recognition, Audio–visual emotion recognition, Audio-visual fusion, Speaker-independent, Feature fusion, Transfer learning, Facial emotion recognition, Speech emotion recognition, Engineering, Mathematics and computing, Neuroscience

## Abstract

Emotion recognition is one of the most important and complex challenges for machines to understand, as most robots and AI agents struggle with human-centric perception and interpretation. Therefore, this paper introduces a novel multimodal emotion recognition system that analyzes emotions through two complementary channels: voice and facial expressions. The proposed approach is evaluated on the RAVDESS and CREMA-D datasets, which consist of acted emotional expressions across multiple discrete emotion categories. Utilizing an advanced multimodal deep feature fusion technique, the system combines handcrafted audio features (e.g., Mel-Frequency Cepstral Coefficients (MFCCs)) with deep visual features extracted from an attention-based VGGFace model. These features are integrated into a unified representation through a hybrid fusion strategy that jointly employs concatenation, cross-attention, gated fusion, and multiplicative fusion mechanisms to capture complementary cross-modal interactions. To ensure a comprehensive and realistic assessment, the model is evaluated under both random-split and strict speaker-independent protocols. On the RAVDESS dataset, the proposed system achieves an accuracy of 95.83% under random-split evaluation and 48.06% ± 9.76% accuracy under speaker-independent Leave-One-Speaker-Out (LOSO) testing, while on the CREMA-D dataset it attains 73.54% accuracy using random splits and 53.12% ± 2.65% accuracy under subject-exclusive speaker-independent 5-fold cross-validation.

## Introduction

Emotion recognition is a human ability that adults acquire through nonverbal cues. Human emotions do not follow fixed patterns or rules that can be directly applied to achieve a result. Rather, human emotions are psychological and physical responses that appear differently across individuals. Each person shows emotions through unique methods, which make their emotional expressions different from others.

Thus, recognizing emotions may be somewhat complex, but adults can intuitively recognize the feelings of others based on their knowledge of many factors, such as how to differentiate between different vocal tones that carry underlying emotions, such as the tone of voice that conveys laughter or sadness. They can also differentiate between various body movements that convey emotions, not just the movements that express joy or disgust, or the facial expressions that convey meanings that indicate a person’s feelings. Recognizing human emotions is relatively easy for individuals, who are able to infer them from speech, tone of voice, facial expressions, body movements, and other forms of body language. The necessity to teach machines emotional understanding through computational methods has emerged because our world now depends on artificial intelligence and robotic systems. The research into machine emotion recognition through computational methods started because of this need.

Emotion recognition research began gradually, and with the remarkable progress of the last decade in the field of artificial intelligence and machine learning, researchers began developing new methods with diverse approaches and varying levels of accuracy and efficiency. This is due to the development of algorithms, deep learning, and increased computing power. The emergence of massive datasets has encouraged researchers to develop accurate emotion recognition models^1^.

Emotion recognition is a crucial and rapidly evolving research area^2^. The future of the smart world depends on communication between humans and intelligent robots, such as smart personal assistants. This makes machine recognition of human emotions a critical issue that must be developed specifically. Machine understanding of humans is essential for communication between them, and it will certainly benefit humans. One of the most important areas where machine understanding of human emotions can help is psychiatry. Emotion recognition may aid in early diagnosis of depression or anxiety disorders, and it can be integrated into Internet of Things (IoT) systems to provide personalized responses. Emotion recognition has become a critical research domain requiring advanced computational frameworks.

Research began to develop this topic gradually, and this development took many stages and forms. The idea of recognizing emotions began by teaching machines how to directly analyze still images of faces. Other research also addressed the idea of teaching machines to analyze voice to obtain sufficient information to recognize emotions. However, these models exhibited many weaknesses in performance, which are considered suboptimal for this domain. Hence, ideas and research emerged about combining models that obtain emotion information from voice and images together to achieve better performance and higher accuracy^3^. This is done by combining information between voice and images to obtain more complex information, creating a more complex model that can infer and predict the correct human emotions with greater accuracy^4^.

Recent research has focused on developing this idea to achieve higher levels of perfection^5^, and extracting features from voice and images has become more complex to achieve the most accurate possible combinational features. Research has concentrated on deep learning models that automatically extract complex features from data using Convolutional Neural Networks (CNN), Long Short-Term Memory (LSTM), and attention networks^6^.

The current research shows that modern models now use multimodal architectures because these systems achieve better performance through their ability to process audio, image, and text data^7^. The model receives enough information through this approach because it uses multiple information channels instead of depending on a single data stream.

The advanced fusion techniques demonstrate effectiveness, yet most current models use only one fusion approach between attention-based and temporal-based methods. The use of a single fusion approach fails to detect all possible relationships between different modal data. The information preservation of simple concatenation does not show any interaction between features, yet attention mechanisms tend to concentrate on important details while ignoring broader patterns. This research fills an existing knowledge gap through the development of a new multi-view fusion system. The model uses four different fusion approaches, which operate independently to create a complete representation of the data through (1) basic feature concatenation, (2) cross-modal attention fusion, (3) gated fusion, and (4) interactive multiplicative fusion. The model combines its four parallel feature streams into one high-dimensional vector. The stacking ensemble classifier processes the unified multi-view representation produced by the fusion architecture, integrating information from multiple audio–visual interaction pathways to support multimodal emotion recognition.

## Background and related works

### Background

Emotion recognition has gradually advanced in complexity and efficiency. The first models implemented for emotion recognition were simple and unimodal. These models relied on a single type of input, such as the unimodal audio model, which analyzes audio, extracts its features, and attempts to identify emotions through them. Other models, such as the unimodal image model, rely solely on extracting features from images and training the model on them to predict and recognize emotions. However, these unimodal models were limited in robustness and generalization^8^. The limitation was primarily due to the inherent ambiguity of emotional cues when observed from a single modality, such as audio or image. It is fundamentally difficult even for ordinary humans to understand the type of emotion in speech alone, without observing facial expressions, body movements, etc.

Therefore, the inefficiency of unimodal models has been emphasized. As a result of advances in artificial intelligence, especially in deep learning, and the emergence of datasets, there has been a shift towards integrated multimodal models that use more than one input channel (audio and images) to improve efficiency and increase the significant features that the model is trained on, producing a robust model capable of correctly recognizing emotions, since most of the features required for the model to perform its task are available^2,5,9^.

### Related works

#### Unimodal emotion recognition (audio-only/visual-only)

Unimodal emotion recognition formed the earliest line of research in affective computing. Audio-only approaches typically relied on prosodic and spectral features such as Mel-Frequency Cepstral Coefficients (MFCCs), chroma representations, or energy-based descriptors, which were later paired with deep learning architectures to improve robustness^8^. Despite these developments, several studies have demonstrated that acoustic features alone exhibit significant limitations when emotional cues are subtle, low-energy, or heavily speaker-dependent^10^.

Similarly, visual-only systems based on still facial images or frame-level CNN encoders struggled with intra-class variability, illumination changes, occlusions, and expression ambiguity. Although deep architectures such as CNNs and 3D-CNNs improved spatial and spatiotemporal modeling, visual-only methods remain sensitive to head pose and lack the contextual depth needed for robust generalization^5,11^.

Taken together, prior unimodal research consistently confirms that emotions rarely manifest strongly in a single modality. Speech encodes prosodic dynamics but omits facial musculature activation, while facial expressions reveal geometry but not intonation. This inherent incompleteness paved the way for multimodal approaches designed to integrate richer, complementary information streams.

#### Multimodal emotion recognition

The shift toward multimodal frameworks stems from the recognition that emotional expression is inherently cross-modal, with audio and visual cues jointly contributing to human affect perception. Numerous studies have demonstrated that combining speech and facial information substantially enhances recognition accuracy and reduces ambiguity, especially under naturalistic or noisy conditions^2,4,7^.

A central component of multimodal systems is visual feature extraction. Transfer learning with CNNs trained on large-scale face identity datasets has emerged as a dominant strategy. Backbone architectures such as VGG-16 and ResNet-50 have consistently shown strong generalization when fine-tuned on emotion datasets^12,13^.

In line with these findings, our work utilizes VGGFace with a ResNet-50 backbone^6,14^, which provides a robust representational space capable of handling facial variability and capturing emotion-specific features.

#### Temporal modeling approaches

Emotional cues unfold dynamically over time, making temporal modeling an essential component of modern emotion recognition pipelines. Long Short-Term Memory (LSTM)-based architectures have been widely explored due to their capacity to encode long-range dependencies in audiovisual sequences. The study in^9^ demonstrated that bi-layer LSTMs combined with multi-head attention significantly improve temporal alignment across modalities.

Parallel research explored Transformer-based architectures, which can attend to multiple temporal positions simultaneously and capture global emotional dynamics^14^. introduced Aural Transformers that effectively model prosodic progression, while^15^ showed that Transformer-based bimodal models outperform traditional recurrent networks in capturing expressive transitions. These temporal models highlight a crucial point: emotion is not static, and effective systems must accommodate the continuous evolution of facial muscle activation, vocal intensity, and rhythm.

#### Fusion strategies in multimodal emotion recognition

Fusion is at the core of multimodal processing and determines how well a model can exploit complementary signals.

- **Early Fusion**:

Early fusion directly concatenates multimodal features into a single representation. Although simple and computationally efficient, it often fails to capture interaction patterns between modalities, especially when modalities encode asynchronous or heterogeneous information^5,16^.

- **Mid-Level Fusion**:

Mid-level, or feature-transformation, fusion attempts to model relationships between modalities by introducing gating or learned projections^12^. showed that gated fusion can dynamically suppress noisy modalities and highlight informative ones, leading to stronger robustness under challenging acoustic or visual conditions.

- **Late Fusion**:

Late fusion aggregates decisions from independently trained unimodal models. Although it provides stability and interpretability, late fusion struggles to capture fine-grained cross-modal interactions. The study in^3^ demonstrated improvements using CNN-based ensembles but noted that decision-level aggregation cannot replace the representational richness of early or mid-level fusion.

Across all three paradigms, a consistent conclusion emerges that no single fusion strategy universally dominates across datasets, noise levels, or modality imbalance conditions. This motivates systems that combine multiple complementary fusion mechanisms rather than relying on one.

#### Attention-based and cross-modal fusion

Attention mechanisms have become central to next-generation multimodal fusion due to their ability to emphasize semantically relevant features. Cross-modal attention, in particular, allows information in one modality to guide feature selection in another. The study in^12^ demonstrated that cross-modal attention significantly enhances emotion recognition in audio-degraded environments. Similarly, the study in^5^ validated that attention-guided alignment improves the interpretability and discriminative power of fused audiovisual features. These findings underscore that attention does not merely combine modalities but orchestrates their interaction in a context-dependent manner, enabling the model to prioritize emotionally salient cues.

#### Ensemble and stacking approaches

Ensemble learning has historically improved robustness in multimodal systems. Tree-based ensembles such as Random Forests and gradient boosting^17,18^, as well as multimodal hierarchical ensembles, demonstrated that combining classifier outputs can reduce variance and enhance generalization^4^. However, most existing ensembles operate on unimodal or single-fusion representations. Applying stacking to multi-view fused features, as performed in our framework, enables the meta-classifier to exploit cross-fusion complementarities—an approach largely unexplored in prior literature.

#### Evaluation protocols in emotion recognition

Recent emotion recognition studies employ a variety of evaluation protocols, reflecting different assumptions about generalization and deployment scenarios. The most commonly adopted approach is the random data split, where samples are randomly divided into training and testing sets. While this protocol is simple to implement and often yields higher reported performance, it does not explicitly control for speaker overlap between training and test partitions. As a result, random splits may unintentionally allow speaker-specific characteristics—such as vocal timbre, facial structure, or expressive style—to be learned during training and reused during testing, leading to inflated performance estimates. To address this limitation, an increasing number of studies advocate for speaker-independent evaluation protocols, such as Leave-One-Speaker-Out (LOSO) or subject-exclusive cross-validation. These protocols explicitly enforce speaker exclusivity between training and test sets, ensuring that all evaluation samples originate from previously unseen speakers. This setting provides a more realistic and challenging assessment of emotion recognition systems, particularly for real-world applications where models are expected to generalize across diverse users rather than adapt to known identities^19–21^.

Several works have highlighted that performance gaps observed between random-split and speaker-independent evaluation can be substantial, underscoring the sensitivity of emotion recognition models to inter-speaker variability. Consequently, speaker-independent protocols are increasingly regarded as a more reliable benchmark for assessing robustness, generalization, and practical applicability. This methodological shift reflects a broader trend in affective computing toward evaluation strategies that prioritize realism and deployment relevance over optimistic upper-bound performance^19–21^.

#### Recent advances in multimodal emotion analysis

Recent multimodal emotion analysis research has expanded beyond classical fusion pipelines toward architectures that explicitly enhance robustness, contextual modeling, and cross-modal interaction quality. A major trend is the emergence of transformer- and diffusion-based models, which address the instability of early-fusion mechanisms and the noise sensitivity seen in tensor-style interactions. For instance, Zhu et al. introduced RMER-DT (Robust Multimodal Emotion Recognition based on Diffusion and Transformers)^22^, a diffusion-transformer framework capable of progressively refining noisy multimodal embeddings and demonstrating that iterative denoising significantly improves recognition consistency in conversational environments. Such findings directly support the motivation behind hybrid fusion systems like ours, which similarly aim to stabilize cross-modal interactions in the presence of heterogeneous input quality.

A complementary direction explores contrastive learning and generative alignment to mitigate modality imbalance—a core challenge in audiovisual emotion recognition. The work of Xiang, Zhu, and Cambria showed that combining audio–visual generation with contrastive objectives strengthens semantic alignment between modalities, reducing contradictions between facial and vocal cues^23^. Similarly, Wang et al. proposed a contrastive-based negative information removal^24^, demonstrating that models benefit from suppressing misleading modality contributions—an idea consistent with the gated and attention-based components in our architecture, which selectively reweight informative cues and suppress noise-driven activations.

Another influential line of research examines adversarial and robust fusion strategies. The RAFT (Robust Adversarial Fusion Transformer) model by Wang et al. introduced adversarial perturbation during training to expose weaknesses in cross-modal fusion layers^25^. This adversarial pressure resulted in more reliable high-level emotional representations. The same principle underlies our decision to combine four heterogeneous fusion paradigms, as multiple parallel fusion pathways mitigate the risk of a single fragile mechanism dominating the representation.

Beyond robustness, several studies emphasize contextual and semantic reasoning. The CIME (Contextual Interaction-based Multimodal Emotion Analysis) framework by Hazarika et al. demonstrated that emotion recognition improves substantially when intermodal interactions incorporate semantic context rather than relying solely on raw features^26^. This perspective aligns with our cross-attention branch, which models directional dependencies between modalities—an approach validated by the improvements seen in modern contextual architectures.

Additionally, a number of works investigate multimodal reliability and missing-modality resilience, a major obstacle for deployment in realistic environments. Zhang et al. introduced a random modality dropout generator, enabling emotion models to maintain performance even when audio or visual channels deteriorate^27^. Likewise, Zhu et al. proposed a client–server behavioral-state system showcasing practical requirements for latency, reliability, and multimodal fallback strategies^28^. These contributions collectively justify incorporating multiple independent fusion pathways in our system, as redundancy increases reliability under imperfect input conditions.

Recent research has also explored advanced feature enhancement and multimodal representation compression. The DNLN (Deformable Non-Local Network) architecture by Chen et al. demonstrated that deformable non-local attention and weighted feature fusion significantly strengthen visual-resolution quality, which is essential when emotion cues depend on micro-expressions^29^. Meanwhile, Wang et al. proposed a tensor decomposition-based multimodal fusion^30^, showing that factorized tensor spaces can retain expressive cross-modal interactions while reducing the instability traditionally associated with full outer-product fusion.

Extending beyond emotion recognition, broader multimodal AI literature provides valuable theoretical grounding. Zhu et al. reviewed multimodal architectures and highlighted challenges in balancing expressive fusion with generalization—precisely the problem addressed by our hybrid multi-view design^31^. Similarly, Zhu et al. (2023) proposed a biologically inspired hierarchical perception framework, demonstrating that structured multi-level integration improves emotional inference^32^; this parallels our stacking-based meta-learning layer, which consolidates diverse fusion outputs into a cohesive decision surface. Even research outside direct emotion recognition—such as knowledge distillation for perception in intelligent transportation systems—demonstrates the broader trend toward combining heterogeneous signals through hierarchical fusion and teacher–student frameworks, reinforcing the relevance of ensemble-like designs such as ours.

These advancements collectively justify the need for the parallel multi-view hybrid fusion architecture proposed in this paper, which operationalizes these principles by combining complementary fusion paradigms—concatenation fusion, gated fusion, cross-attention fusion, and interactive multiplicative fusion—followed by a stacking meta-learner to consolidate their strengths.

#### Research gap and novelty of the proposed approach

Despite substantial recent progress in multimodal emotion recognition, the literature continues to exhibit several critical limitations, particularly in studies conducted on benchmark datasets such as the Ryerson Audio-Visual Database of Emotional Speech and Song (RAVDESS). Most recent architectures—whether based on transformers, diffusion models, or contrastive cross-modal alignment—typically adopt a single fusion paradigm and optimize it for robustness, contextual modeling, or noise suppression. While these approaches improve representation quality, they do not investigate how multiple complementary fusion mechanisms may jointly capture distinct and non-overlapping interaction patterns between audio and visual modalities. As a result, existing systems generally model cross-modal interactions from only one structural perspective, such as attention-guided fusion, gated enhancement, or tensor-based multiplicative interactions. This design choice restricts the expressive capacity of the fused feature space and overlooks the possibility that different fusion hypotheses encode complementary emotional cues.

Moreover, although recent studies have introduced robustness-oriented strategies such as modality dropout and adversarial perturbation, these methods do not explore parallel multi-branch early fusion, where each branch embodies a fundamentally different interpretation of how modalities should interact. In addition, the literature shows limited investigation of stacking-based meta-learning applied to multimodal fused representations. Most existing systems rely on either single-branch early fusion or transformer-driven late fusion, without integrating heterogeneous fused embeddings into a higher-level ensemble classifier capable of learning decision boundaries that are not expressible by any individual fusion strategy. Consequently, higher-layer decision integration—an important mechanism for improving robustness and generalization—remains largely underexplored.

A second and equally important gap concerns evaluation methodology. While many multimodal emotion recognition studies report strong performance under random data splits, fewer works adopt speaker-independent evaluation protocols, which are crucial for assessing real-world generalization. Recent investigations have demonstrated that random splits may significantly overestimate performance due to speaker overlap between training and test sets, motivating the adoption of stricter subject-exclusive evaluation schemes. Majkowski and Kołodziej^19^, for instance, reported an accuracy of 36.2% on the RAVDESS dataset under a subject-independent SS-70/10/20 protocol for speech emotion recognition, highlighting notable performance degradation compared to speaker-dependent evaluation. Similarly, Portal et al.^20^ conducted a comprehensive benchmarking of Transformer-based models for speaker-independent speech emotion recognition, emphasizing the sensitivity of modern architectures to inter-speaker variability. Chakhtouna et al.^21^ further showed that speaker and gender dependencies play a substantial role in cross-linguistic emotion recognition performance, reinforcing the necessity of speaker-independent validation.

To address these methodological and architectural gaps, we propose a parallel multi-view hybrid fusion framework in which four conceptually distinct early-fusion mechanisms—concatenation, gated fusion, cross-attention, and multiplicative fusion—are executed simultaneously. Each branch captures a different modality-interaction hypothesis, producing a set of complementary fused embeddings. These embeddings are subsequently integrated through a stacking ensemble meta-classifier, enabling the model to learn high-level decision boundaries that cannot be captured by any single fusion paradigm alone. This design yields a richer, redundancy-aware multimodal representation and improves robustness under modality noise and inter-speaker variability.

**Fig. 1:**
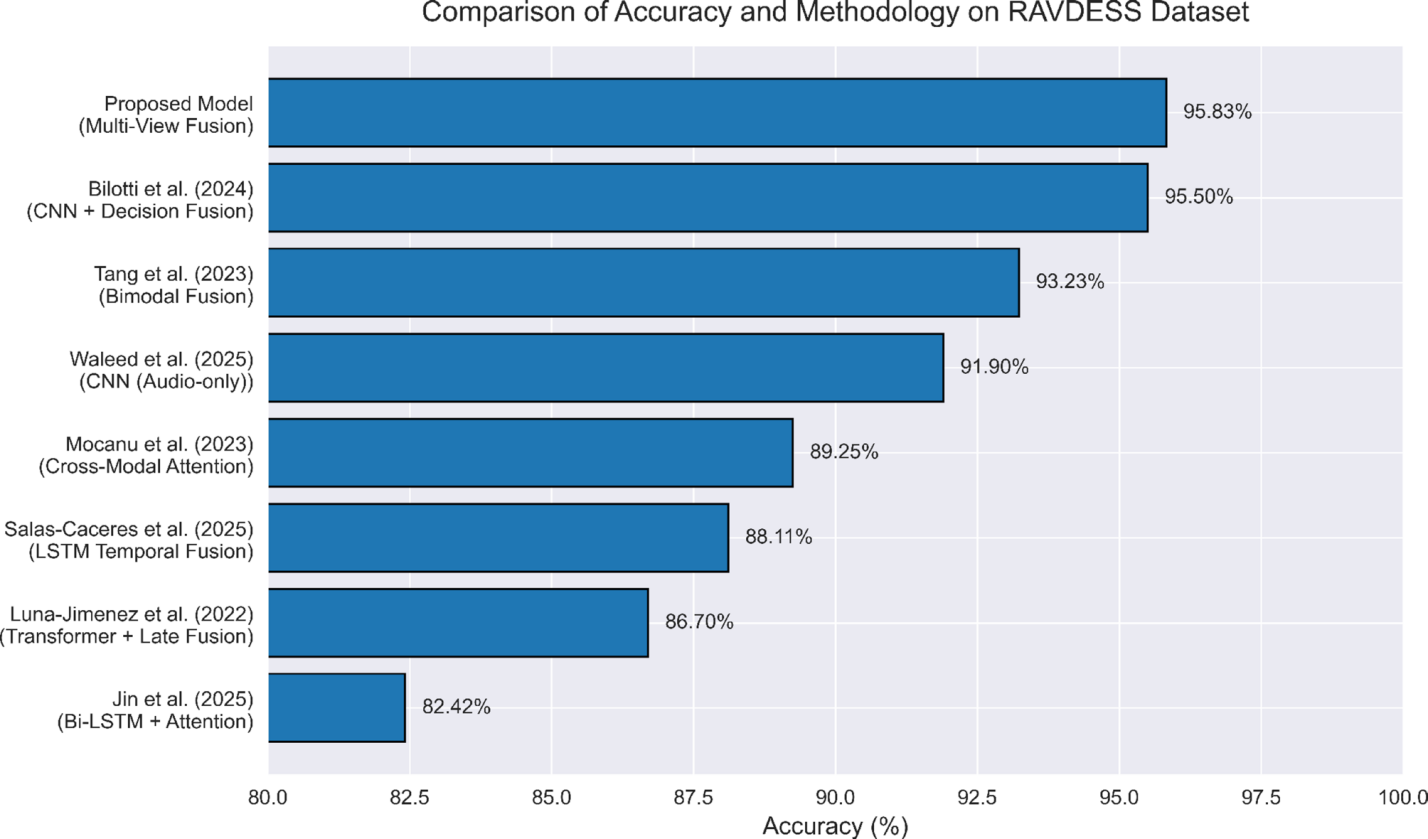
Accuracy comparison of recent RAVDESS emotion recognition models under non-speaker-independent evaluation protocols, including the proposed multi-view fusion approach.

Table 1 summarizes key RAVDESS-based emotion recognition studies by outlining the specific methodological designs and architectures adopted in each work. Figure 1 visually compares the reported classification accuracies of these approaches on the RAVDESS dataset, highlighting the relative performance trends across different fusion strategies and model architectures. Jin et al.^9^ implemented a bi-layer LSTM equipped with multi-head attention to model temporal audio–visual dependencies, achieving 82.42% accuracy under a subject-exclusive 5-fold cross-validation setup. Luna-Jiménez et al.^6^ combined Aural Transformer encoders for speech with facial action unit descriptors, enabling complementary modeling of spectral and facial muscle activity; their system was evaluated using subject-exclusive 5-fold cross-validation (5-fold CV). Salas-Cáceres et al.^13^ incorporated sequential audio–visual fusion to explicitly capture temporal emotional trajectories, reporting 88.11% accuracy using an 80/20 train–test split. Mocanu et al.^5^ introduced a cross-modal attention module coupled with deep metric learning to encourage alignment between audio and visual embeddings, though the evaluation protocol was not reported in the publication. Waleed et al.^33^ applied a CNN-based spectrogram encoder for speech emotion recognition and evaluated their model using an 80/20 stratified split, achieving 91.90%. Tang et al.^4^ investigated both feature-level and decision-level bimodal fusion using CNN backbones; however, the authors did not specify their evaluation protocol. Bilotti et al.^3^ developed a CNN-based multimodal fusion system that integrates spatial and acoustic cues, reporting an accuracy of 95.50%, with the evaluation protocol similarly not documented. For comparison, the proposed multi-view hybrid system processes each audio–visual sample through four distinct early-fusion mechanisms—concatenation, gated fusion, cross-attention, and multiplicative fusion—before aggregating them via a stacking ensemble classifier. Our proposed framework is evaluated on the RAVDESS dataset under both random-split and strict speaker-independent settings, providing a more transparent and comprehensive performance assessment. Under a stratified random split, the proposed model achieves an accuracy of 95.83%, demonstrating strong performance relative to existing RAVDESS-based emotion recognition systems. More importantly, under speaker-independent Leave-One-Speaker-Out (LOSO) evaluation, the model attains an average accuracy of 48.06% ± 9.76%, while maintaining statistically validated improvements over unimodal and single-fusion baselines. This dual evaluation strategy highlights the robustness of the proposed multi-view fusion framework and directly addresses a critical methodological gap in prior multimodal emotion recognition research.

**Table 1:** Comparison of recent RAVDESS-based emotion recognition studies under non-speaker-independent evaluation protocols.

Author(s) (Year)	Modalities	Methodology/Features	Accuracy (%)
Jin et al. (2025)^9^	Audio + Video	Bi-Layer LSTM + Multi-Head Attention	82.42%
Luna-Jimenez et al. (2022)^6^	Audio + Video	Aural Transformers + Action Units (Late Fusion)	86.70%
Salas-Caceres et al. (2025)^13^	Audio + Video	Audiovisual Fusion with LSTM for Temporal Dynamics	88.11%
Mocanu et al. (2023) ^5^	Audio + Video	Cross-Modal Attention Fusion + Deep Metric Learning	89.25%
Waleed et al. (2025) ^33^	Audio	Convolutional Neural Network (CNN)	91.90%
Tang et al. (2023) ^4^	Audio + Video	Bimodal Feature and Decision-Level Fusion	93.23%
Bilotti et al. (2024) ^3^	Audio + Video	CNN-based Feature and Decision-Level Fusion	95.50%
Our Proposed Model	Audio + Video	Multi-View Fusion (Concatenation, Attention, Gated, Multiplicative)	95.83%

## Methodology

**Fig. 2:**
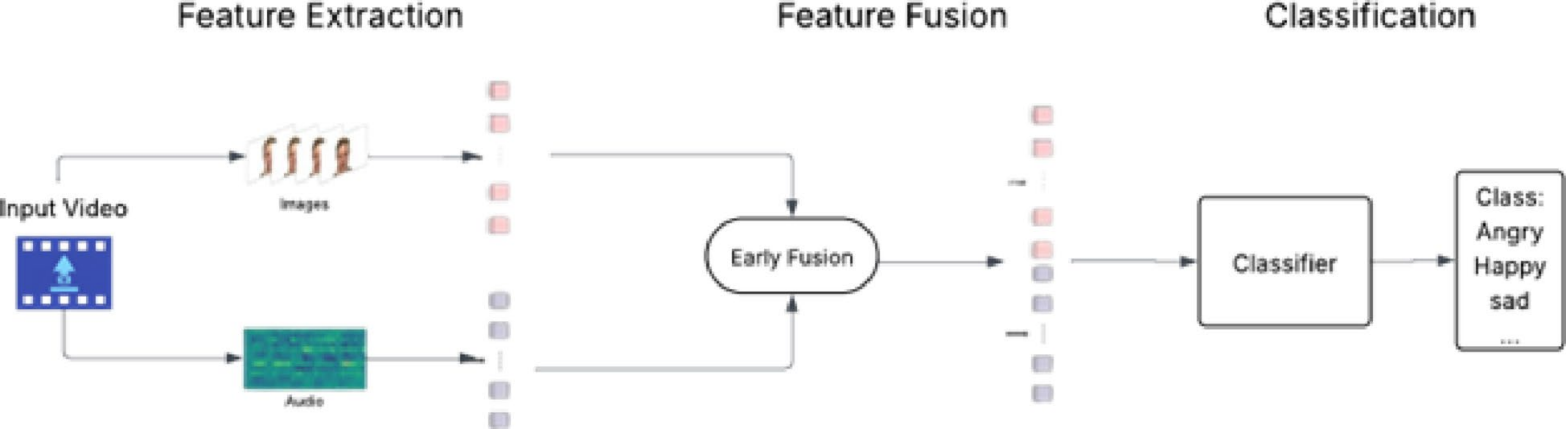
Block diagram of the proposed multimodal emotion recognition system.

**Fig. 3:**
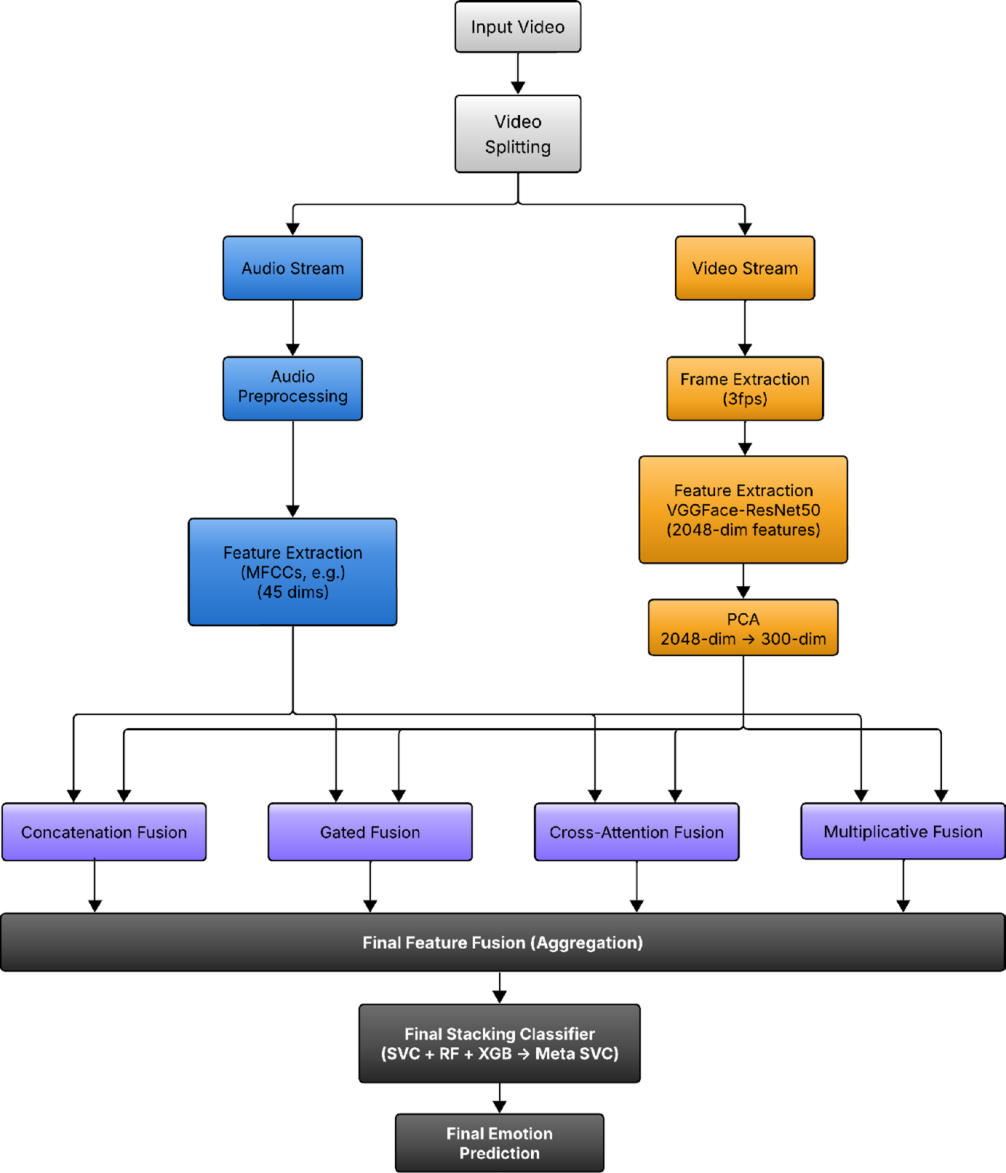
Overview of the proposed multimodal emotion recognition pipeline.

The multimodal model consists of two main branches and three stages, as shown in Fig. 2 and the detailed system pipeline in Fig. 3^5,13,14^. The two main branches are speech emotion recognition and facial emotion recognition. Each branch goes through three main stages: feature extraction, feature fusion, and modeling. Each branch operates independently through a sequence of well-defined stages, including preprocessing, modality-specific feature extraction, and the generation of intermediate representations.

In the audio branch, MFCC-based acoustic features are extracted following standard preprocessing steps, while the visual branch processes video frames using a VGGFace–ResNet50 feature extractor, followed by dimensionality reduction through principal component analysis (PCA). Once both modalities are processed, the system transitions into the multimodal fusion stage.

At this point, four complementary early-fusion strategies are applied in parallel—concatenation, gated fusion, cross-attention, and multiplicative fusion—each capturing different aspects of the interaction between audio and visual representations. The outputs of these branches are then aggregated into a unified feature vector, as depicted in Fig. 3, forming the input to the final stacking ensemble classifier. This hierarchical architecture enables the model to integrate heterogeneous cues across modalities and produce a robust prediction for the emotion label associated with the input video.

### Dataset

#### RAVDESS dataset

In this study, we used the Ryerson Audio-Visual Database of Emotional Speech and Song (RAVDESS) dataset^1,6,9^, one of the most well-known datasets in the field of emotion recognition. The RAVDESS dataset is a publicly available and open-source dataset that contains three modalities (audio-visual, video-only, and audio-only) and two vocal types (speech and song)^2^. Each subset includes eight emotion categories: calm, neutral, happy, sad, angry, fearful, surprised, and disgusted. In our study, we used the audio-visual (AV) data and only the speech part, excluding the song recordings, to train our model^14^. This part of the dataset includes 24 actors (twelve males and twelve females), each performing 60 different videos divided into eight categories, with a total of 1440 videos. The clips range in duration between 2.99 and 5.31 s, as summarized in Table 2. where the actors vocalize two lexically matched statements in a neutral North American accent^14^. Each video has a unique label that encodes the content of the video, which will be used later in the classification process.

#### CREMA-D dataset

In addition to RAVDESS, we also employ the Crowd-sourced Emotional Multimodal Actors Dataset (CREMA-D)^34^, a large-scale dataset specifically designed for multimodal emotion recognition research. CREMA-D contains audio-visual recordings of professional actors expressing emotional content under diverse recording conditions, making it suitable for evaluating emotion recognition systems in more perceptually variable scenarios. The dataset includes recordings from 91 actors (48 male and 43 female) with a wide age range (20–74 years). Each actor performs multiple utterances corresponding to a set of predefined emotional categories, including angry, disgust, fear, happy, neutral, and sad. The emotional expressions are conveyed through spoken sentences, resulting in a total of 7,442 audio-visual clips. A summary of the main characteristics of the CREMA-D dataset used in this study, including the number of actors, samples, modalities, and emotion categories, is provided in Table 3.

**Table 2:** Summary of the RAVDESS dataset.

Attribute	Description	Percentage Distribution (%)
Emotions	8 classes (Neutral, Calm, Happy, Sad, Angry, Fearful, Disgust, Surprised)	~ 13.30% for each of the seven emotional classes, ~ 6.70% for the Neutral class.
Gender	24 actors (12 male, 12 female)	50% male, 50% female.
Intensity	2 levels (Normal, Strong). Note: Neutral has ‘Normal’ intensity only.	Balanced between Normal/Strong for all non-neutral emotions.
Modality	Audio and Video	100% Multimodal
Total Samples	1440 video files (96 Neutral, 192 for each of the other seven emotions)	-

**Table 3:** Summary of the CREMA-D dataset.

Attribute	Description	Distribution
Emotions	6 classes (Angry, Disgust, Fear, Happy, Neutral, Sad)	Non-uniform distribution across emotion classes
Actors	91 actors (48 male, 43 female)	~ 52.7% Male, ~ 47.3% Female
Age Range	20–74 years	Broad age diversity
Utterances	Spoken sentences performed by professional actors	Multiple utterances per actor
Annotation	Crowd-sourced emotion labels	Each sample was annotated by multiple raters.
Modality	Audio and Video	100% Multimodal
Total Samples	7,442 audio-visual recordings	–

### Data preparation and splitting

**Fig. 4:**
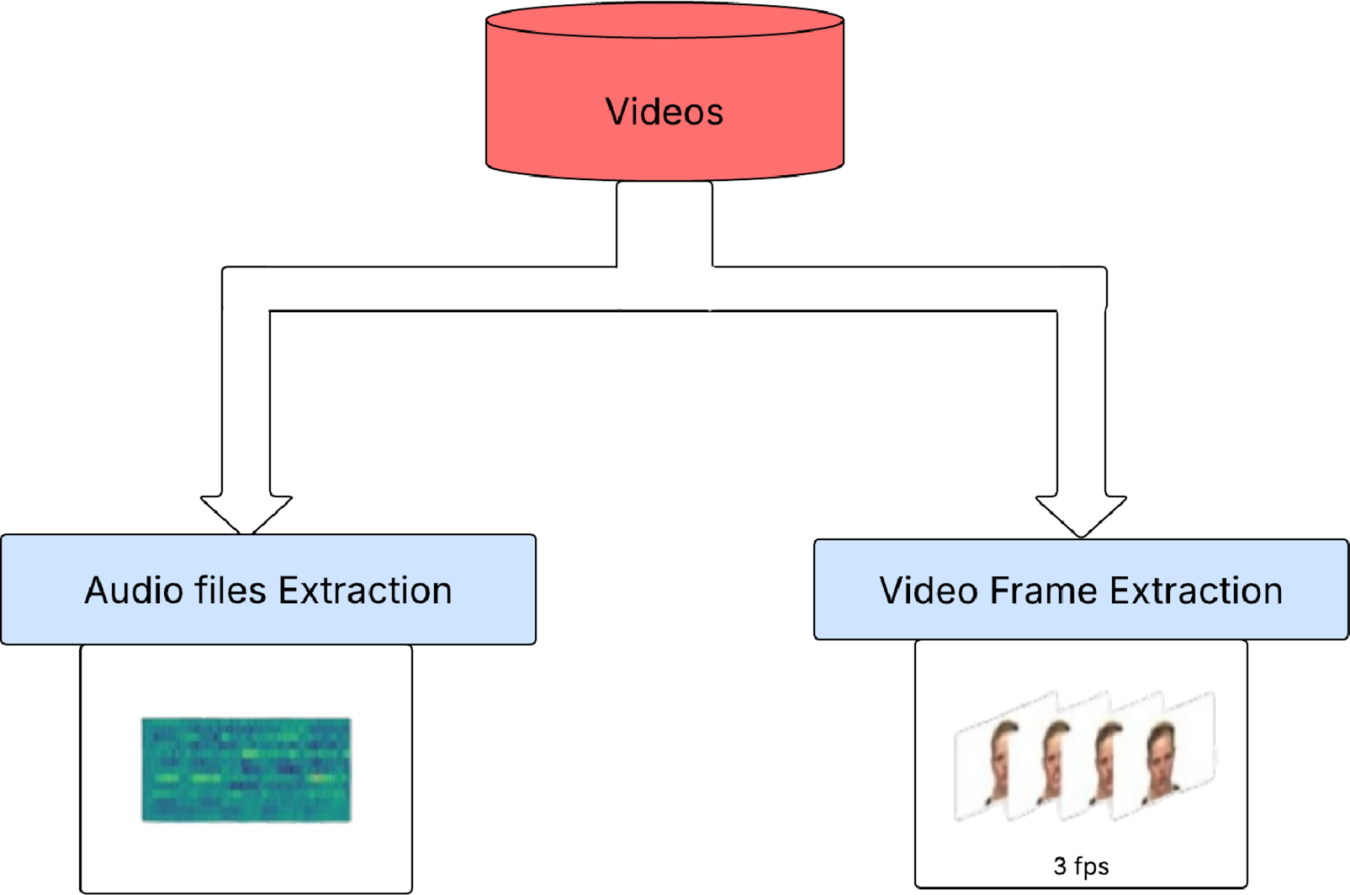
Data preparation process diagram.

As shown in Fig. 4, each video sample is processed through separate audio and visual pipelines to prepare the datafor multimodal feature extraction.

#### Audio file extraction

In this step, we extracted the audio tracks from each video in the datasets. Since the recordings are provided in video format (e.g.,.mp4), the embedded audio streams were separated to enable independent processing of speech signals, as illustrated in Fig. 4. The audio was extracted from the videos using the MoviePy Python library, which is commonly used for video processing. We used it to extract the audio from each video. The extracted audio files were saved in Waveform Audio File (WAV) format to be used in the feature extraction stage^16,35^. Each extracted audio file retained the same unique identifier as its corresponding video, preserving the original metadata and emotion label. This consistent labeling strategy ensures accurate alignment between audio and visual modalities during multimodal learning. In total, one audio file was generated for each audio-visual sample, resulting in a one-to-one correspondence between video recordings and extracted speech signals.

#### Video frame extraction

In parallel with audio processing, visual information was prepared by extracting video frames from each audio-visual recording, inserting them into the top branch in Fig. 2 and performing the three operations on them^6,9,12,14^. The extraction was performed using the OpenCV library, which is used for video processing and splitting videos into a set of frames^5,13^, a setting empirically selected to balance temporal coverage and computational efficiency. Preliminary experiments with lower sampling rates (e.g., 2 FPS) resulted in a noticeable degradation in recognition performance, indicating insufficient visual detail for effective emotion representation. Consequently, the 3 FPS configuration was adopted throughout all experiments.

### Feature extraction

This is the first stage through which the video data from the dataset passes after being separated into image and audio files. It is the foundation for our final model, and it is essential to build a good model to ensure the most effective and meaningful extraction of features from the audio or visual components.

#### Audio feature extraction

The primary task at this stage is to convert the extracted audio files into a set of features that represent the audio files and that our model can understand and train on. The process of extracting features from the audio file was performed using the Librosa library^36^, a widely used library for audio and music processing that has numerous applications in speech recognition and audio feature extraction. We used this library for extracting audio features from the audio file. It was designed to include several steps that are applied to the audio file to extract the features. It consists of:

- Loading the Audio Signal: First, the audio file is loaded using librosa.load, which produces a time-series array and sets a sampling rate of 22.05 kHz for all files. We applied this standardization to ensure that features are uniform across different audio files. Standardizing audio inputs and extracting time-frequency features using Librosa has proven effective in optimizing speech emotion recognition models^8^.
- Feature Calculation: A variety of features are then extracted to capture different acoustic properties of the speech signal. The mean value of each feature across the entire duration of the audio clip is calculated to produce a single representative value for that feature. The selected features are MFCCs, Zero Crossing Rate (ZCR), Spectral Centroid, Spectral Bandwidth, Spectral Rolloff, and Root Mean Square (RMS):
- Mel-Frequency Cepstral Coefficients (MFCCs): 40 MFCCs are extracted, which serve as the most effective spectral representation for sound power analysis and speech recognition applications that model human vocal characteristics. MFCCs are the most common and effective spectral features for speech emotion recognition^10,37^, as demonstrated by Baruah and Banerjee^35^, who achieved 78.50% accuracy on RAVDESS using MFCC-based representations.
- Zero-Crossing Rate (ZCR) measures the number of times the signal transitions from positive to zero and then to negative or from negative to zero and then to positive. The ZCR helps distinguish between voiced sounds like vowels and unvoiced sounds like fricatives.
- The Spectral Centroid feature determines the central point of the sound spectrum, which shows where the most dominant frequencies exist. The perceived brightness of a sound relates to this feature.
- Spectral bandwidth measures the distance between the spectral centroid and the surrounding frequency range to determine signal frequency distribution.
- Spectral rolloff indicates the frequency point where 85% of the total spectral power resides. The feature helps users identify different sound textures.
- Root Mean Square (RMS) Energy feature enables the calculation of signal amplitude root mean square values, which represent audio loudness levels.
- Feature Aggregation: Finally, all the extracted features (the 40 MFCCs plus the five other acoustic features) are concatenated into a single, flat numerical array. This results in a final feature vector of length 45 for each audio file. Similar preprocessing and feature aggregation pipelines were adopted in previous RAVDESS-based studies^16^.

#### Frame feature extraction

**Fig. 5:**
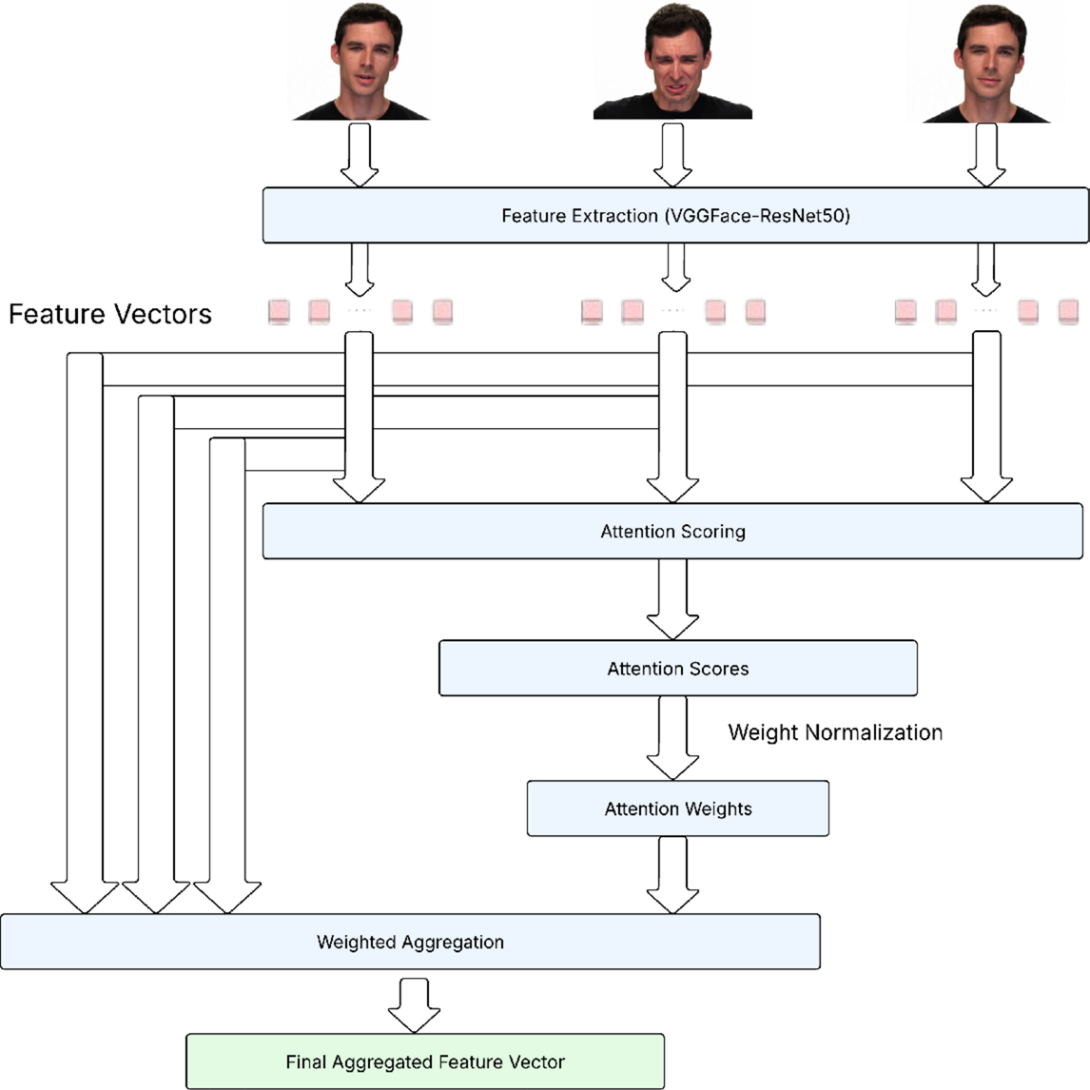
Attention-based aggregation process for extracting the final aggregated facial feature vector from multiple video frames. The facial photographs displayed are sample frames obtained from the publicly available RAVDESS dataset^1^.

At this stage, the primary goal is to obtain high-quality spatial features to ensure effective representation of facial expressions. This is one of the most important stages for our model.

In our proposed model, we used the VGGFace pre-trained model with the ResNet-50 architecture to extract visual features^14,17,18^. This pre-trained model has learned on large-scale face recognition datasets and learned highly discriminative facial representations from millions of facial images, making it well-suited for our model.

In this study, the model was used without its top classification layers and configured with average pooling, enabling the extraction of 2048-dimensional deep feature embeddings from the final convolutional layer for each frame.

The configuration maintains deep generalizable facial descriptors while removing identity and classification-specific layers.

Before passing the frames into the feature extractor, we will perform these operations:

- Resizing the frames to 224 × 224 pixels to fit the input size required for ResNet50 architectures.
- Convert the frames into numerical tensors.
- Preprocessing the array of numerical tensors to align our input with the normalization and scaling parameters used during the model’s original training.

Once the deep features were extracted for each frame, they were aggregated into a single video-level representation using a custom attention-based aggregation mechanism shown in Fig. 5. Attention-based fusion mechanisms have been proven effective in emphasizing emotionally salient frames while reducing redundancy^12,38^. This mechanism was implemented through a weighted feature combination through three key steps:

- Attention Scoring: Each frame feature vector is passed through a Dense layer with a tanh activation to compute an attention score, indicating the importance of that frame.
- Weight Normalization: The attention scores are normalized using a Softmax function along the frame dimension to ensure that all weights sum to one.
- Weighted Aggregation: A weighted sum of frame feature vectors is computed, emphasizing frames with stronger emotional relevance while suppressing neutral or less informative ones.

The output is a single 2048-dimensional vector representing the overall emotional content of the video.

### Feature fusion

This stage represents the main pillar for the success of the core idea of our multimodal model, which is fusing the features of each data type to obtain a single vector of combined features that the model is trained on. The model requires proper preparation at this stage to achieve its best training performance, which leads to successful results. Similar to Zhao et al. and Mocanu et al^5,39^. The fusion process works to extract common and distinct representations between modalities, which results in improved emotion recognition accuracy.

#### Data preparation and preprocessing

For each video, the extracted audio and visual features are first passed to a preparation and preprocessing stage prior to fusion. In this stage, the audio descriptors and the frame-based visual representations are aligned at the video level and organized as two separate unimodal inputs corresponding to the same emotional instance.

The prepared feature sets are then normalized using standardization to ensure comparable scales between modalities and to prevent bias during multimodal interaction. This step is essential for stabilizing learning and enabling effective integration of heterogeneous audio and visual features.

Given the high dimensionality of the visual representations, Principal Component Analysis (PCA) is subsequently applied to the video-level visual features to reduce redundancy and computational complexity while preserving the most discriminative information. The resulting normalized and compact audio–visual representations are finally fed into the multi-view fusion mechanisms described in Sect. 3.4.2.

#### The multi-view fusion model

The development of a strong multimodal representation required us to create an advanced multi-view fusion system. The system contains four separate fusion methods that operate independently of each other. The multi-view approach enables the model to detect various aspects of cross-modal relationships through its ability to handle both basic feature relationships and intricate non-linear patterns. The model generates a unified feature vector through the combination of outputs from its parallel strategies. This architecture takes the preprocessed audio and video features as two separate input branches.

##### Simple concatenation fusion

This is the most direct method of early fusion. The audio and reduced-dimensionality video feature vectors are simply concatenated end-to-end.

$$\:{\mathrm{F}}_{\mathrm{c}\mathrm{o}\mathrm{n}\mathrm{c}\mathrm{a}\mathrm{t}}=[{\mathrm{V}}_{\mathrm{P}\mathrm{C}\mathrm{A}}\:;{\mathrm{A}}_{\mathrm{s}\mathrm{c}\mathrm{a}\mathrm{l}\mathrm{e}\mathrm{d}}]$$

where $\:{\mathrm{V}}_{\mathrm{P}\mathrm{C}\mathrm{A}}$ denotes the visual feature vector after dimensionality reduction, and $\:{\mathrm{A}}_{\mathrm{s}\mathrm{c}\mathrm{a}\mathrm{l}\mathrm{e}\mathrm{d}}$ represents the normalized audio feature vector. The operator $\:\left[\cdot\:;\cdot\:\right]$ denotes feature concatenation.

As in Tang et al. and Bilotti et al^3,4^., this method preserves all original information but does not explicitly model any interactions between the modalities.

##### Cross-modal attention fusion

Inspired by the success of attention mechanisms in sequence modeling, this technique follows the principle demonstrated by Das et al^38^., enabling the model to weigh the importance of features from one modality with respect to the other in a cross-modal context.

Both audio and video inputs are first projected into a shared latent space of 128 dimensions using dense layers with rectified linear unit (ReLU) activation. This projection serves to align the feature representations into a comparable space, facilitating effective interaction between modalities. The chosen dimensionality provides a balance between representational capacity and computational efficiency, where higher dimensions may capture richer interactions at the cost of redundancy and increased complexity, while lower dimensions may limit the ability to model meaningful cross-modal relationships.

Following this alignment, a MultiHeadAttention layer is employed to model cross-modal dependencies^9,40^. In this configuration, the audio features act as the query, while the video features serve as both key and value, producing an attended video representation conditioned on audio. The process is then symmetrically repeated with the roles reversed, allowing the model to capture bidirectional interactions between modalities. The parameters of the projection and attention layers remain fixed during this process, and the resulting attention-enhanced representations are concatenated to form the final fused feature vector.

##### Gated fusion

This method introduces a gating mechanism to regulate the contribution of each modality to the final fused representation. As in Liu et al., Zhao et al., and Kumar et al^17,41,42^., the approach applies a structured weighting scheme to balance the influence of audio and visual features.

A dense layer with a sigmoid activation is applied to both the audio and video inputs, producing a gating value for each modality. These values are concatenated and passed through a SoftMax layer, generating two normalized weights, α (for audio) and β (for video), such that α + β = 1.

The original feature vectors are then scaled by their respective weights and concatenated:

$$\:{\mathrm{F}}_{\mathrm{g}\mathrm{a}\mathrm{t}\mathrm{e}\mathrm{d}}=[{\upalpha\:}.{\mathrm{A}}_{\mathrm{s}\mathrm{c}\mathrm{a}\mathrm{l}\mathrm{e}\mathrm{d}}\:;\:{\upbeta\:}.{\mathrm{V}}_{\mathrm{P}\mathrm{C}\mathrm{A}}]$$

where $\:\alpha\:$ and $\:\beta\:$ denote the modality weighting coefficients assigned to the audio and visual features, respectively.

##### Interactive multiplicative fusion

This approach is designed to explicitly model the complex multiplicative interactions between audio and visual features, following the general formulation in Liu et al^43^..

First, both audio and video feature vectors are projected into a shared latent space of 256 dimensions using Dense layers. This projection ensures dimensional compatibility between modalities and enables effective interaction at a feature level. The selected dimensionality provides sufficient capacity to capture higher-order cross-modal relationships while avoiding excessive expansion that may introduce redundancy and increased computational cost.

Next, an element-wise multiplication is performed between the projected vectors. This operation forms the core of the multiplicative fusion mechanism, generating a joint representation in which each element reflects the interaction between corresponding audio and visual features. The parameters involved in the projection remain fixed during this process.

Finally, a dropout layer (rate = 0.3) is applied to reduce overfitting, followed by a ReLU activation function to introduce non-linearity into the fused representation.

#### Final aggregation and feature extraction

The outputs from all four fusion techniques (concatenation, cross-attention, gated, and multiplicative fusion) are concatenated into a single feature vector.

$$\:{\mathrm{F}}_{\mathrm{f}\mathrm{i}\mathrm{n}\mathrm{a}\mathrm{l}}=[{\mathrm{F}}_{\mathrm{c}\mathrm{o}\mathrm{n}\mathrm{c}\mathrm{a}\mathrm{t}}\:;{\mathrm{F}}_{\mathrm{a}\mathrm{t}\mathrm{t}\mathrm{e}\mathrm{n}\mathrm{t}\mathrm{i}\mathrm{o}\mathrm{n}}\:;{\mathrm{F}}_{\mathrm{g}\mathrm{a}\mathrm{t}\mathrm{e}\mathrm{d}}\:;{\mathrm{F}}_{\mathrm{m}\mathrm{u}\mathrm{l}}\:]$$

where $\:{\mathrm{F}}_{\mathrm{c}\mathrm{o}\mathrm{n}\mathrm{c}\mathrm{a}\mathrm{t}}$, $\:{\mathrm{F}}_{\mathrm{a}\mathrm{t}\mathrm{t}\mathrm{e}\mathrm{n}\mathrm{t}\mathrm{i}\mathrm{o}\mathrm{n}}$, $\:{\mathrm{F}}_{\mathrm{g}\mathrm{a}\mathrm{t}\mathrm{e}\mathrm{d}}$, and $\:{\mathrm{F}}_{\mathrm{m}\mathrm{u}\mathrm{l}}$ represent the feature representations obtained from different fusion strategies, which are combined to form the final feature vector.

Inspired by Fan et al^44^., multiple fusion outputs were concatenated to form a 1202-dimensional feature vector before classification, as summarized in Table 4.

**Table 4:** Composition of the final feature vector after applying the four fusion techniques.

Fusion Component	Output Description	Output Dimensionality
Concatenation Fusion	Direct sequential concatenation of the audio (45-dim) and PCA-reduced video (300-dim) features.	345
Cross-Attention Fusion	A vector resulting from a cross-attention mechanism, where each modality learns to weigh the importance of the other.	256
Gated Fusion	A weighted combination of the original audio and video features, where the model learns dynamic gates for each modality.	345
Interactive Multiplicative Fusion	A vector that captures non-linear interactions between features via element-wise multiplication in a projected space.	256
Total	Sum of the dimensions of the four components.	1202

### Classification model training and evaluation

The multi-view fused features served as input for a robust machine learning pipeline, which trained and evaluated a classification model. The system needs to identify emotion classes through these complete feature sets. The proposed system uses a stacking classifier to combine different base models, which have proven effective for improving robustness in multimodal emotion recognition systems^2–5^.

#### Data preparation

Data preparation is a critical step to ensure valid and reproducible model evaluation and to support reliable generalization across experimental settings^9,13,14^. The following preprocessing stages are applied to the fused feature representation:

1. **Data Loading and Partitioning**: The fused feature set is loaded and organized into an input feature matrix and a corresponding target label vector by excluding non-informative identifiers.
2. **Label Encoding**: Categorical emotion labels are converted into numerical representations to enable supervised learning.
3. **Feature Scaling**: Feature normalization is performed using standardization, with scaling parameters learned exclusively from the training set and subsequently applied to validation and test data to prevent information leakage.

#### The stacking ensemble model

We implemented in the proposed model an ensemble model called a stacking classifier; this model is a multi-level architecture where one final classifier learns from the predictions of the other base models in the previous level^2^.

- Level 0: Base Classifiers: In our proposed study, three distinct machine learning algorithms are used as base estimators, which serve as powerful and diverse base classifiers. Their role is to make initial predictions on the data.

1. Support Vector Classifier (SVC): A strong classifier to determine the best possible hyperplane for class separation. The RBF kernel function was used for this classification^16^.
2. Random Forest Classifier (RF): An ensemble of 300 decision trees that corrects for overfitting by averaging the results.
3. XGBoost Classifier (XGB): This algorithm represents a highly efficient and powerful gradient boosting system, which achieves top results in numerous competitive events.

- Level 1: The Meta-Classifier (Final Estimator): The predictions from the three base classifiers are not the final output. Instead, they are used as input features for a final meta-classifier.

1. Our final classifier is a linear support vector classifier (SVC).
2. The base models generated output probabilities instead of definite class labels for their predictions. The meta-classifier receives a more detailed and complex input from this approach, which enables it to produce a more accurate final prediction.

#### Model training and evaluation

- **Training**: The proposed stacking ensemble classifier was trained independently on both the RAVDESS and CREMA-D datasets using their corresponding training partitions. For each dataset, model training was performed on multimodal fused features derived from audio and visual representations, allowing the classifier to learn discriminative emotion patterns from complementary modalities. The same model architecture and hyperparameter configuration were maintained across both datasets to ensure a fair and consistent performance comparison.
- **Evaluation Protocol**: To assess the generalization capability of the proposed framework under speaker-independent conditions, different evaluation protocols were adopted for each dataset according to its structural characteristics. For the RAVDESS dataset, a Leave-One-Speaker-Out (LOSO) protocol was employed, where all samples from one speaker were held out for testing while the remaining speakers were used for training, ensuring strict speaker-independent evaluation. For the CREMA-D dataset, a subject-exclusive 5-fold cross-validation protocol was applied, in which speakers were partitioned into five mutually exclusive folds such that no speaker appeared simultaneously in training and testing sets. This protocol provides a balanced and robust assessment of cross-speaker generalization while maintaining sufficient training data in each fold. In addition, a random stratified 72/8/20 split (RS) was used for both datasets to evaluate model behavior under conventional experimental conditions where speaker overlap between training and testing sets is permitted, enabling direct comparison with prior works reported under similar settings.
- **Evaluation Metrics**: Performance was quantified using standard classification metrics, including overall accuracy as well as class-wise precision, recall, and F1-score. These metrics provide a comprehensive view of both global classification performance and per-emotion behavior, particularly in the presence of class imbalance.
- **Confusion Matrix and Classification Report**: Confusion matrices were employed to examine detailed class-level prediction patterns and to identify systematic confusions among emotion categories. In addition, classification reports were used to summarize precision, recall, and F1-score values for each emotion class, enabling a fine-grained evaluation of model performance without bias toward dominant categories.

### Implementation and reproducibility

All experiments were conducted under a strict speaker-independent evaluation setting, as described in Sect. 3.5.3. Experiments were performed on two benchmark emotion recognition datasets, namely RAVDESS and CREMA-D, which were selected to evaluate the robustness of the proposed multimodal fusion framework across datasets with different scales and emotion label granularities.

#### Model parameters

As summarized in Table 5, key hyperparameters were selected based on empirical validation experiments and the observed behavior of multimodal fusion architectures:

- **Dimensionality Reduction**: PCA was applied to reduce the 2048-dimensional visual feature vectors to 300 components. This value was chosen because it preserves more than 90–95% of the meaningful variance while removing high-dimensional noise that often destabilizes early-fusion mechanisms^45^. Lower PCA values (< 200) led to loss of fine-grained spatial information, whereas higher values (> 400) increased redundancy and computational cost without any performance gain^46^.
- **Regularization Strategy**: A dropout rate of 0.3 was adopted across the fusion branches to improve generalization. Smaller rates (< 0.2) were insufficient to suppress overfitting caused by the interaction between audio and visual embeddings, while larger rates (≥ 0.4) interfered with convergence and degraded the model’s ability to retain discriminative cross-modal cues. The intermediate value of 0.3 provided the most stable training behavior and the highest validation macro F1-score^47^.
- **Base Learners**: The stacking classifier used SVC as its base learner with C = 10 and an RBF kernel. The larger value of C enabled better separation between emotion classes, especially when distinguishing between neutral and calm emotions and fearful and sad emotions. The RBF kernel maintained its position as the best choice because it delivered superior results to linear kernels when working with audio-visual embeddings that created non-linear patterns^48^.
- **Ensemble Models**: The ensemble components were configured with 300 estimators to enhance prediction stability and reduce variance, especially for underrepresented emotion classes, while maintaining reasonable computational efficiency^49^. For the XGBoost classifier, a maximum tree depth of 5 was adopted to mitigate overfitting; deeper trees led to unstable validation behavior, whereas shallower trees failed to capture sufficient semantic structure from the fused facial–acoustic features. A learning rate of 0.1 was selected to ensure stable convergence and consistent generalization across folds and datasets^50^. All ensemble hyperparameters were fixed and consistently applied across both RAVDESS and CREMA-D experiments to ensure fair comparison and reproducibility.
- **Meta-Learner Configuration**: At the meta-learning level, the stacking classifier employed a linear SVC trained on the class-probability outputs of the base learners (i.e., probability-based stacking). The use of a linear decision boundary at this stage was intentionally chosen to limit model complexity and prevent overfitting to noisy or redundant base-level predictions. This design encourages the meta-learner to exploit complementary information across fusion branches in a stable and interpretable manner, thereby improving robustness under speaker-independent evaluation settings.

**Table 5:** Hyperparameter Configuration.

Component/Model	Parameter	Value Used
Principal Component Analysis (PCA)	n_components	300
Cross-Attention	projection_dim	128
	num_heads	8
Multiplicative Fusion	projection_dim	256
	dropout_rate	0.3
Support Vector Classifier (SVC)	kernel	RBF
	C	10
	probability	TRUE
Random Forest	n_estimators	300
Extreme Gradient Boosting (XGB)	n_estimators	300
	max_depth	5
	learning_rate	0.1
Meta-Learner: SVC	kernel	linear
	probability	TRUE

#### Frame sampling policy

To ensure consistent temporal representation and full reproducibility, a uniform frame sampling strategy was adopted for all video samples. Specifically, frames were extracted at a fixed rate of three frames per second from each video, providing balanced temporal coverage while avoiding excessive redundancy and computational overhead. This policy preserves both low- and high-intensity emotional expressions across the video timeline without relying on heuristic key-frame selection. All extracted frames were resized to a fixed spatial resolution and processed identically, ensuring that variability in frame count or duration does not introduce bias into the visual feature extraction stage.

#### Training settings

All experiments were conducted using fixed random seeds to ensure deterministic behavior and reproducibility across runs. Feature normalization was applied using standardization, with scaling parameters estimated exclusively from the training data to prevent information leakage. For each LOSO iteration/CV fold, PCA and feature scaling were fitted exclusively on the training speakers and subsequently applied to the held-out test speakers. Visual features were reduced in dimensionality using PCA prior to fusion to mitigate redundancy in high-dimensional representations. During fusion, dropout regularization was applied to interaction branches to control model complexity and improve generalization. Training was performed using speaker-independent protocols as defined in the experimental setup, ensuring that no identity information leaked between training and evaluation phases.

### Computational complexity analysis

A computational complexity analysis is conducted to evaluate the efficiency, scalability, and practical feasibility of the proposed multimodal framework. The system comprises distinct processing stages, each contributing differently to the overall computational cost. The audio-processing branch is computationally lightweight, as MFCC extraction relies primarily on short-time Fourier transform operations with approximately $\:O\left(Tlog\right(T\left)\right)$, where denotes the number of audio frames. In contrast, the visual-processing branch represents the dominant computational component of the system. Feature extraction using the VGGFace–ResNet50 backbone requires a forward pass through a deep convolutional architecture, which scales roughly with $\:O(n\cdot\:d)$, where is the number of sampled frames (3 fps in our setting) and $\:d=2048$, corresponding to the dimensionality of the penultimate representation. Although computationally intensive, this stage is executed once during preprocessing and therefore does not directly impact inference-time latency. To reduce dimensionality and mitigate computational overhead in subsequent fusion stages, PCA is employed to project the 2048-dimensional feature vector into a 300-dimensional subspace. The training cost of PCA scales with $\:O\left(N{d}^{2}\:\right)$, where denotes the number of training samples, but inference requires only a matrix–vector multiplication of cost $\:O(d\cdot\:300)$. This dimensionality reduction significantly lowers the computational burden of subsequent fusion and classification stages while preserving discriminative information.

The fusion stage introduces varying levels of computational cost depending on the employed mechanism. Concatenation fusion incurs minimal overhead, scaling linearly with feature dimensionality. Gated fusion adds lightweight element-wise modulation operations and remains computationally efficient. In contrast, cross-attention fusion requires the computation of interaction matrices between modalities, with approximate complexity $\:O\left({d}^{2}\:\right)$, making it more demanding. Multiplicative fusion, based on element-wise interactions between projected feature vectors, introduces only linear complexity $\:O\left(d\right)$, as it operates through dimension-wise multiplication without constructing higher-order representations. This makes it significantly more efficient while still capturing direct cross-modal interactions.

The classification stage is comparatively efficient, as it operates on compact fused representations rather than high-dimensional raw features. Classical classifiers such as SVC, Random Forest, and XGBoost exhibit low inference-time complexity, primarily dominated by kernel evaluation or tree traversal on reduced feature vectors, resulting in fast prediction times suitable for real-time or near-real-time applications.

Beyond theoretical complexity, the practical computational footprint of the framework remains manageable. The model does not rely on large end-to-end multimodal transformers with extensive parameter counts; instead, it leverages pre-extracted features and modular fusion strategies operating on reduced-dimensional representations. Consequently, training time remains relatively moderate compared to end-to-end multimodal architectures, as it is primarily influenced by feature extraction and classifier training rather than iterative deep multimodal optimization. Additionally, the modular design supports scalability, allowing individual components (audio, visual, and fusion branches) to be executed or optimized independently depending on available computational resources.

Overall, the computational analysis indicates that visual feature extraction constitutes the primary computational bottleneck, while fusion mechanisms introduce varying levels of overhead depending on their complexity. Notably, the most computationally intensive operations are largely associated with preprocessing, whereas inference can be performed more efficiently due to dimensionality reduction and the use of lightweight classifiers. These observations suggest that the proposed framework provides a reasonable balance between representational capacity and computational cost, supporting its potential feasibility for real-world multimodal emotion recognition applications, although further optimization may be required for strict real-time deployment scenarios.

## Results and discussion

### Model performance and discussion

The performance of the proposed multi-view fusion framework was systematically evaluated on two widely used benchmark datasets, namely RAVDESS and CREMA-D, under both conventional random split and strict speaker-independent evaluation protocols to ensure a comprehensive and fair assessment across datasets and speaker conditions.

#### RAVDESS dataset

##### Performance under random split

**Fig. 6:**
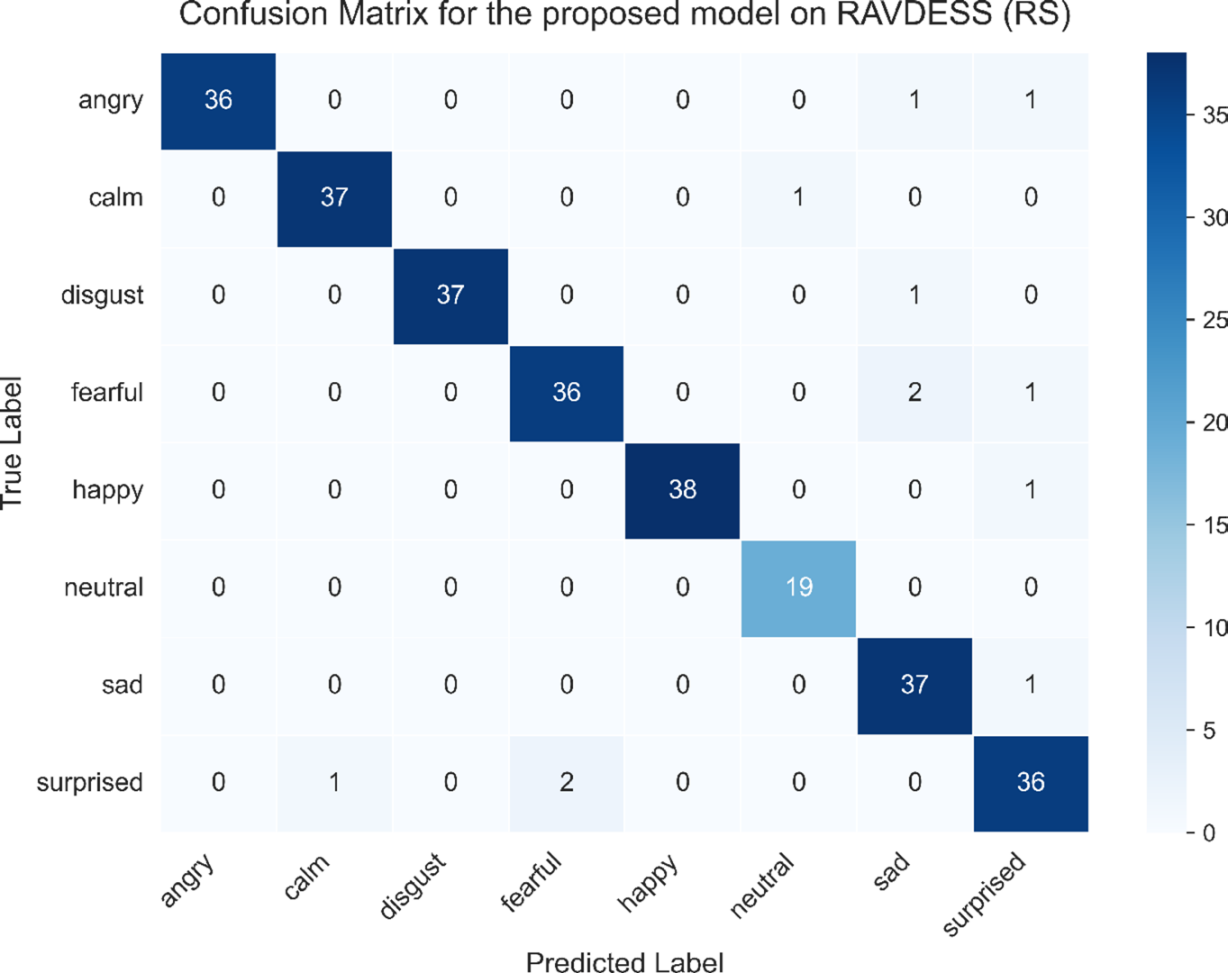
Confusion matrix of the proposed model on RAVDESS dataset under Random Split (RS) evaluation.

**Fig. 7:**
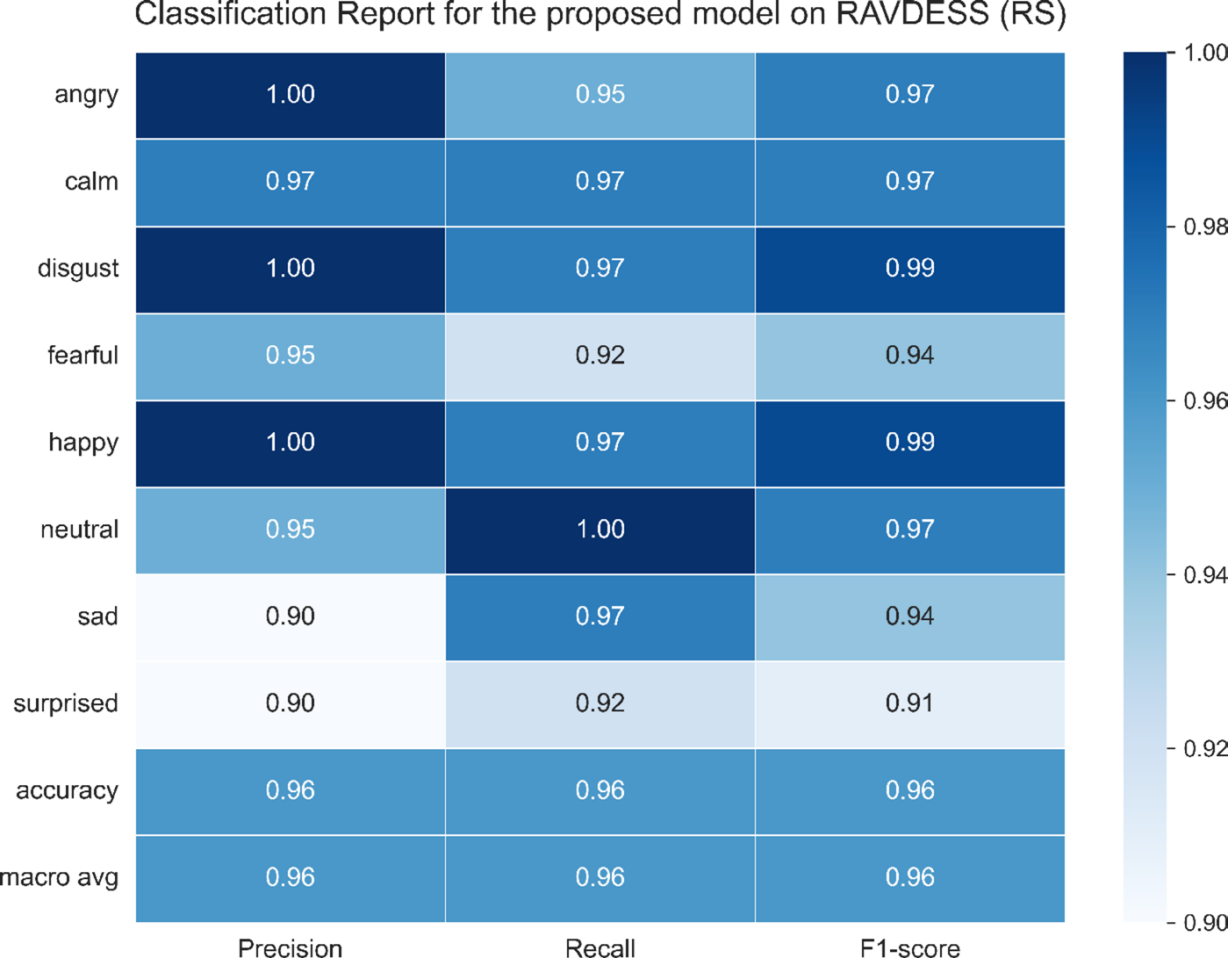
Classification report for the proposed model showing precision, recall, F1-score, and accuracy for each emotion class on RAVDESS dataset under Random Split (RS) evaluation.

Under the conventional random 72/8/20 split (RS), the proposed model achieved a high test accuracy of 95.83% on RAVDESS dataset, indicating strong discriminative capability when speaker overlap is allowed between training and testing sets. This result is consistent with recent studies on the RAVDESS dataset that report high performance under similar evaluation settings, such as the work of Jin and Zai, where a bi-layer LSTM with multi-head attention achieved strong emotion recognition performance across multiple categories^3,5,12^. Figure 6 illustrates the confusion matrix of the proposed model, providing a detailed view of its classification behavior across the eight emotion classes. The dominance of diagonal entries indicates strong alignment between true and predicted labels, with high recall observed for several classes, including Happy (38 correctly classified samples) and Calm (37 correctly classified samples). The model also correctly identified all samples of the neutral class, further highlighting its effectiveness in modeling subtle emotional expressions despite limited data availability. Minor misclassifications were primarily observed between acoustically and emotionally similar categories, such as Calm and Neutral, Fearful and Sad, and Happy and Surprised. These confusions are consistent with known overlaps in prosodic characteristics, including pitch variation, energy level, and vocal tension, commonly shared among these emotion pairs. Overall, the results confirm that the proposed framework achieves strong and stable discrimination across emotion categories under conventional evaluation settings, with errors confined to semantically related classes. The classification report, presented in Fig. 7, demonstrates consistently high classification performance across all emotion categories under the random split evaluation protocol. The proposed model achieved F1-scores of 0.99 for both the Disgust and Happy classes, indicating strong discriminative capability for highly expressive emotional states. Notably, the neutral class—despite being a minority category—was classified with a high F1-score of 0.97, reflecting the model’s robustness to moderate class imbalance and its ability to capture subtle, low-intensity emotional cues.

##### Performance under leave-one-speaker-out

**Fig. 8:**
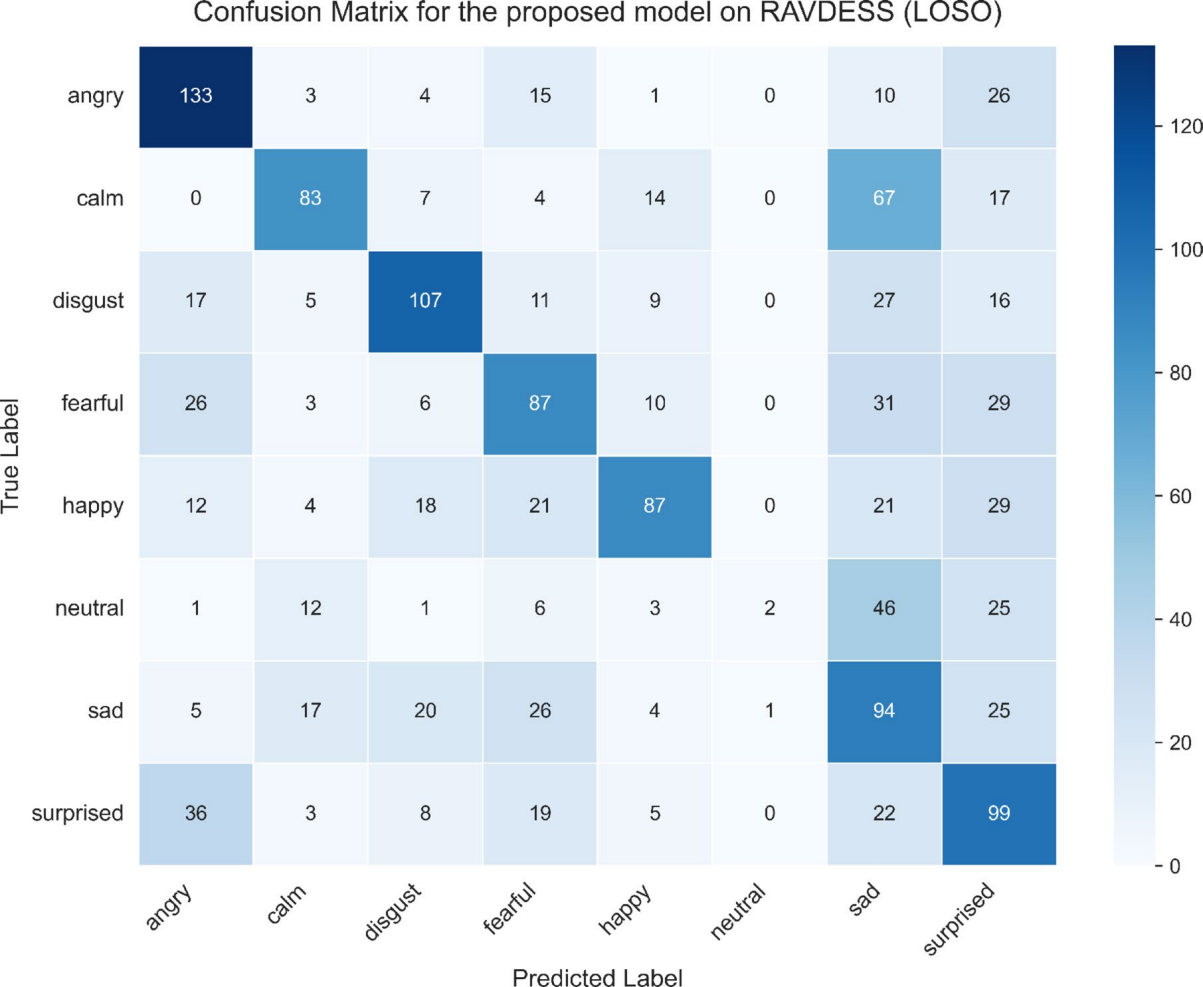
Confusion matrix of the proposed model on RAVDESS dataset (LOSO).

**Fig. 9:**
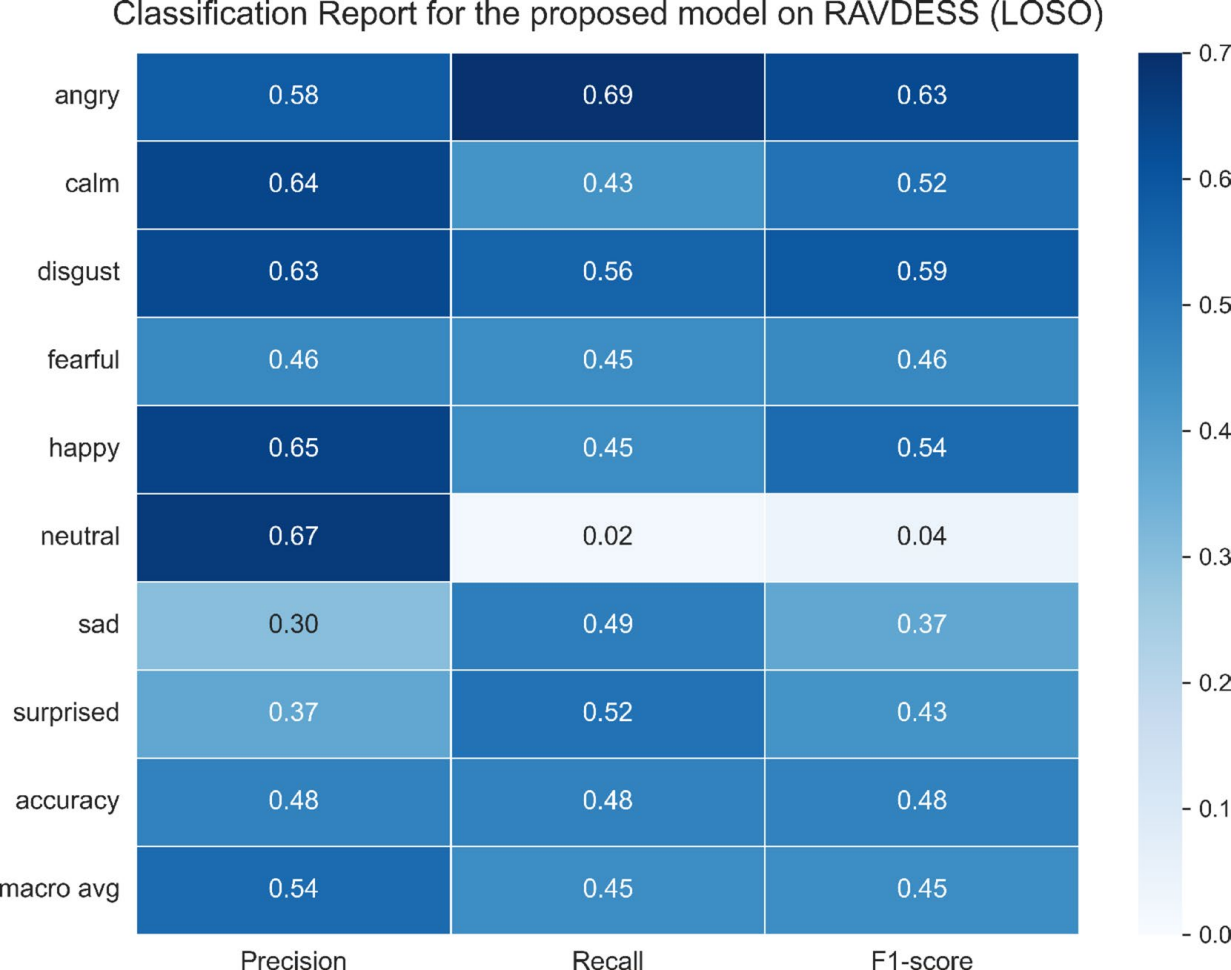
Classification Report of the proposed model on RAVDESS dataset (LOSO).

To further assess generalization under strict speaker-independent conditions, a Leave-One-Speaker-Out (LOSO) evaluation protocol was applied on the RAVDESS dataset. Under this setting, the proposed multi-view fusion model achieved an average accuracy of 48.06% ± 9.76%, which is substantially lower than the RS performance but consistent with prior speaker-independent emotion recognition studies on acted datasets^19^. The noticeable gap between RS and LOSO results reflects speaker-specific variability rather than methodological deficiencies, highlighting the intrinsic difficulty of cross-speaker emotion recognition. Despite this challenge, the model maintained stable performance well above chance level, indicating that the learned audio–visual representations capture meaningful emotional cues that partially generalize to unseen speakers. As shown in the confusion matrix and classification report (Figs. 8 and 9, respectively), expressive emotions such as Angry, Disgust, and Happy achieved the highest F1-scores (up to 0.63), demonstrating stronger cross-speaker consistency. In contrast, the neutral class is relatively under-represented compared to more expressive emotions in the RAVDESS dataset, resulting in fewer training samples and limiting the model’s ability to learn robust, speaker-invariant representations. Moreover, neutral expressions often lack salient emotional cues and exhibit substantial overlap with low-intensity affective states such as Sad, Calm, and Surprised. This proximity in the audio–visual feature space increases ambiguity and leads to frequent misclassifications, particularly under speaker-independent evaluation protocols where baseline expression patterns vary significantly across speakers.

##### Overfitting analysis and generalization

**Fig. 10:**
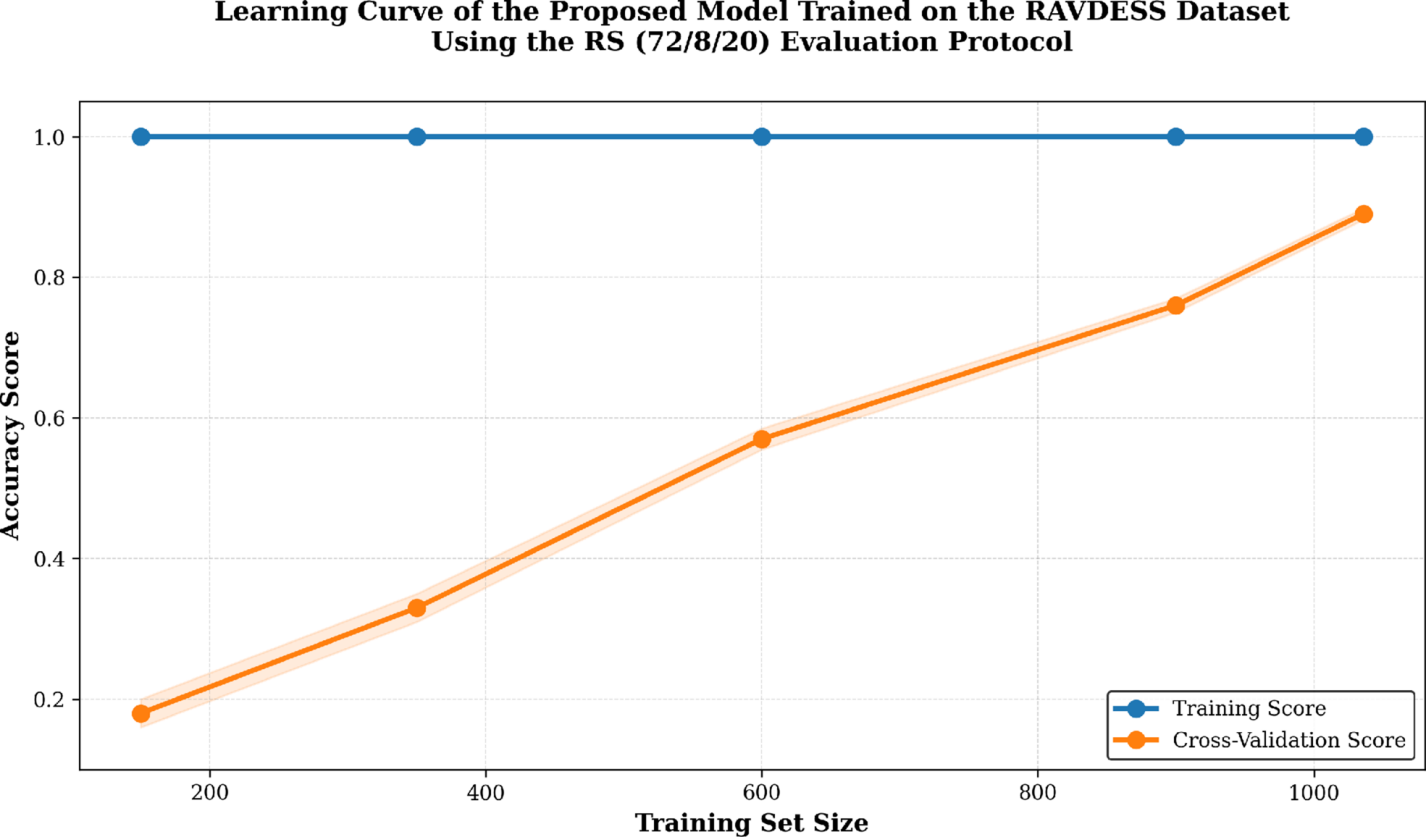
Learning curve of the proposed model trained on the RAVDESS dataset using the RS (72/8/20) evaluation protocol.

Figure 10 presents the learning curve of the proposed model under the random split (RS) evaluation protocol. The training accuracy remains consistently high and approaches saturation, indicating that the model is able to fit the training data effectively under conditions where speaker overlap is present. In contrast, the validation accuracy increases steadily as the training set size grows, reaching the highest accuracy at full data utilization. This upward trend suggests that the model continues to benefit from additional training samples, with no clear indication of early performance saturation.

A noticeable gap between training and validation performance is observed, which may be attributed to the relatively high representational capacity of the model in relation to the dataset size. However, the gradual improvement of validation accuracy, together with the absence of performance degradation as more data are introduced, suggests that this gap is not solely a result of uncontrolled overfitting. Instead, it likely reflects the difference between fitting overlapping speaker distributions in the training set and generalizing to held-out samples within the same distribution.

These observations indicate that, under the RS setting, the model benefits from the availability of overlapping speaker distributions, which facilitates fitting the training data while supporting gradual improvements in validation performance. At the same time, the persistent gap between training and validation accuracy suggests that further improvements are more likely to depend on increased data diversity rather than additional model complexity.

**Fig. 11:**
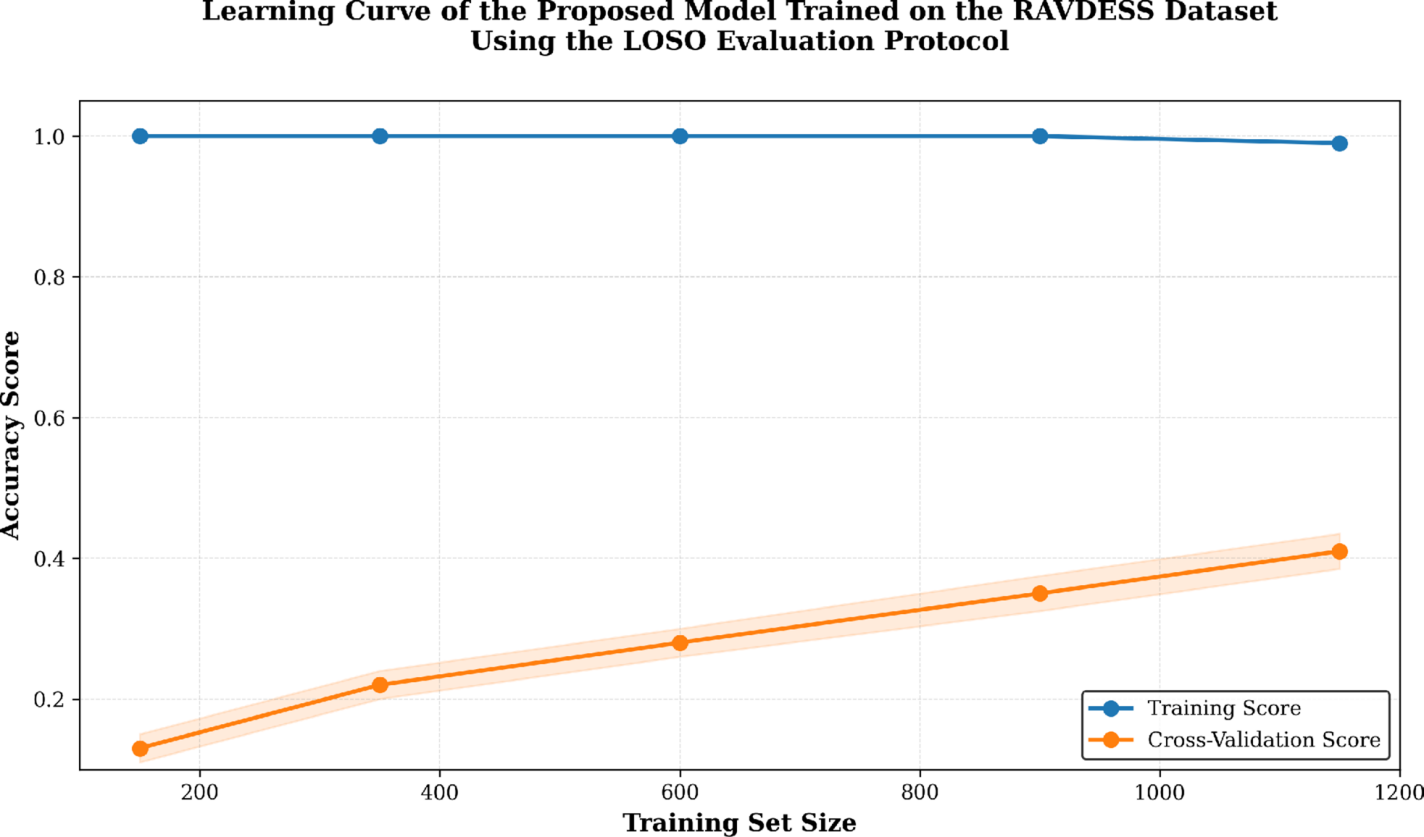
Learning curve of the proposed model trained on the RAVDESS dataset using the LOSO evaluation protocol.

Figure 11 illustrates the learning curve of the proposed model under the Leave-One-Speaker-Out (LOSO) evaluation protocol. The training accuracy remains consistently high, indicating that the model is able to effectively capture patterns within the training speakers. In contrast, the test accuracy on unseen speakers starts at a lower level and increases gradually as the training set grows, suggesting that the model benefits from increased exposure to diverse speaker characteristics.

A clear gap between training and test performance is observed, which is expected under speaker-independent evaluation settings. This gap can be attributed primarily to inter-speaker variability, where differences in vocal characteristics, facial dynamics, and expression styles introduce additional challenges that are not present in random split scenarios. Rather than indicating uncontrolled overfitting, this behavior appears to reflect the inherent difficulty of generalizing across unseen speakers. Similar trends have been reported in prior speaker-independent emotion recognition studies, where performance degradation is commonly observed when models are evaluated on speakers not seen during training^19–21^. The gradual improvement of the test curve as more data are introduced suggests that the model is able to capture partially speaker-invariant emotional representations, although this process remains limited by the available dataset size and diversity. This interpretation is further supported by the observed quantitative gap, where performance decreases from approximately 95% on samples from seen speakers to 48.06% ± 9.76% on unseen speakers. Such a difference highlights the contrast between intra-speaker discrimination and cross-speaker generalization, indicating that the primary challenge lies in modeling variability across speakers rather than fitting the training data.

#### CREMA-D dataset

##### Performance under random split

**Fig. 12:**
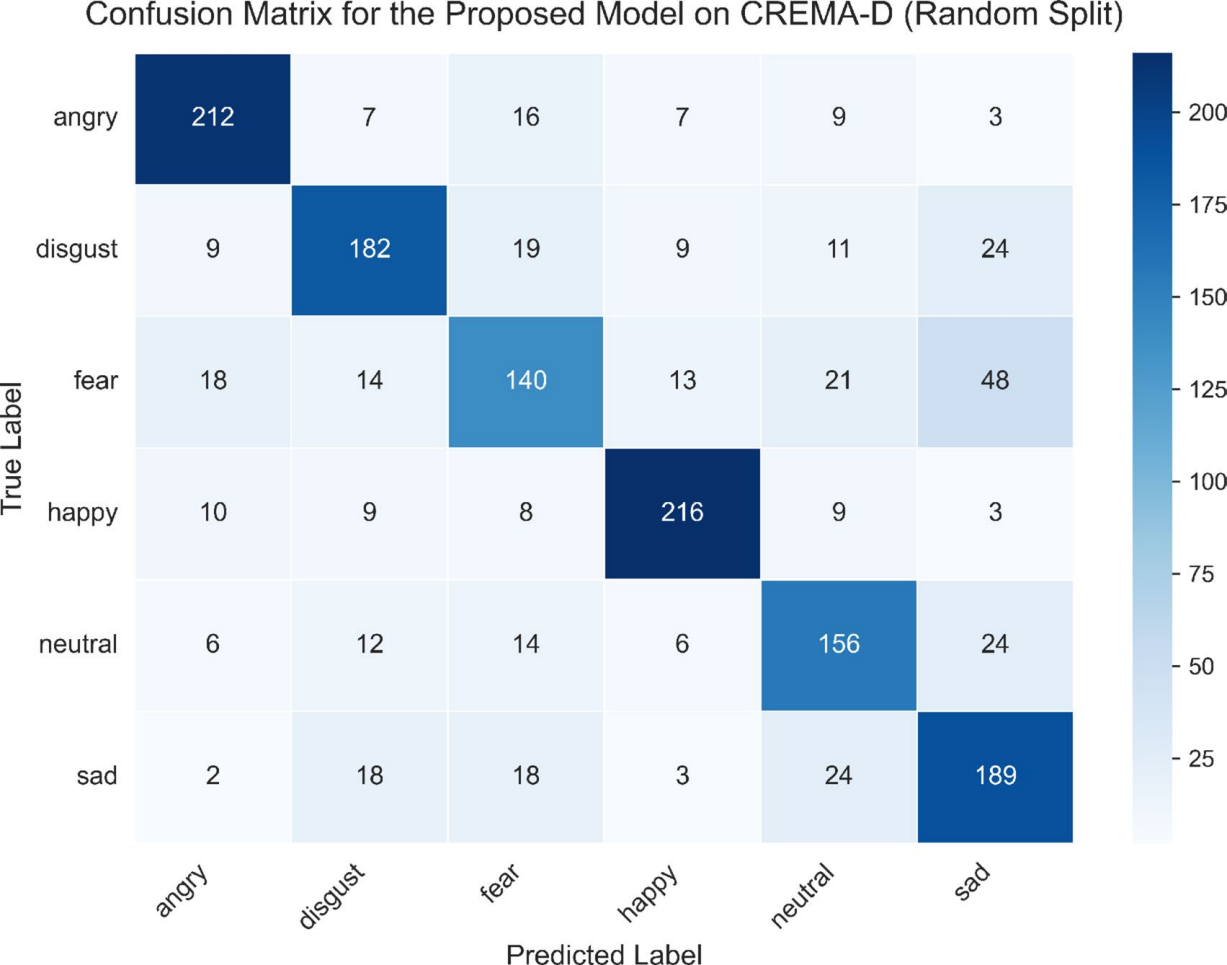
Confusion matrix of the proposed model on CREMA-D dataset under Random Split (RS) evaluation.

**Fig. 13:**
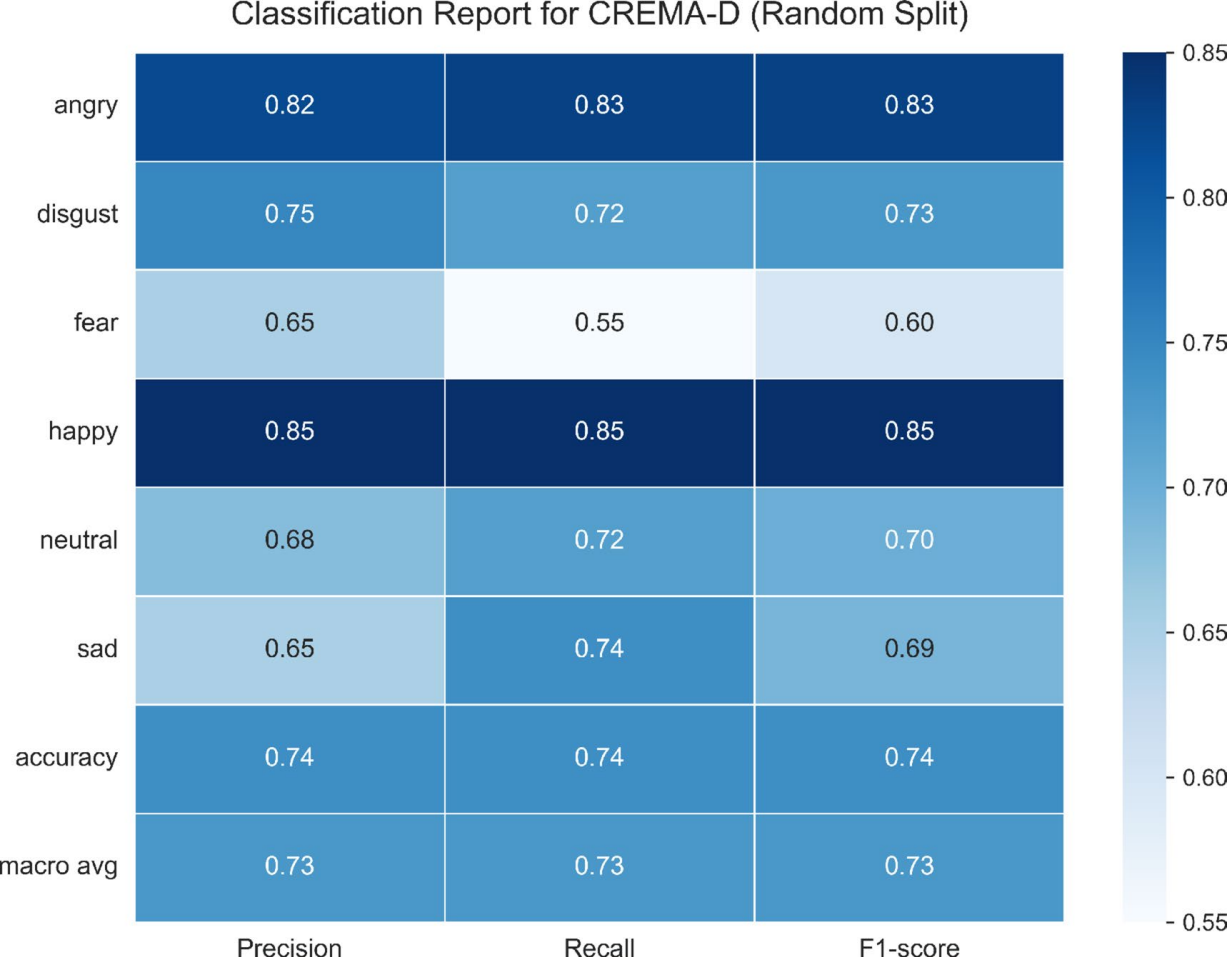
Classification report for the proposed model showing precision, recall, F1-score, and accuracy for each emotion class on the CREMA-D dataset under Random Split (RS) evaluation.

Under the conventional random 72/8/20 split (RS), the proposed multi-view fusion model achieved a test accuracy of 73.54% on the CREMA-D dataset. Compared to RAVDESS, this lower performance is expected due to the increased variability in speaker identity, recording conditions, and expressive styles inherent to CREMA-D, which presents a more challenging emotion recognition scenario. Nevertheless, the obtained accuracy demonstrates the model’s ability to generalize effectively when speaker overlap between training and testing sets is allowed. Figure 12 illustrates the confusion matrix for CREMA-D under the RS evaluation protocol, providing detailed insight into the model’s class-wise behavior across the six emotion categories. Strong diagonal dominance is observed for highly expressive emotions such as Angry (212 correctly classified samples) and Happy (216 correctly classified samples), indicating robust recognition of high-arousal affective states. The sad class also exhibits strong performance with 189 correctly classified samples, reflecting reliable modeling of low-energy emotional cues. Moderate confusion is primarily observed between acoustically related emotion pairs, particularly Fear and Sad, Neutral and Sad, and Disgust and Fear, which is consistent with known overlaps in vocal characteristics such as pitch contour, spectral energy distribution, and articulation patterns. The neutral class, which is inherently less expressive and often overlaps with neighboring affective states, shows comparatively lower recall, highlighting the intrinsic difficulty of modeling subtle emotional expressions in speaker-diverse datasets. The classification report presented in Fig. 13 further confirms these observations. The model achieves strong F1-scores for Angry (0.83) and Happy (0.85), demonstrating reliable discrimination of high-intensity emotions. More challenging categories such as Fear (0.60) and Neutral (0.70) yield lower F1-scores, reflecting the ambiguity and acoustic similarity associated with these emotions in CREMA-D. The macro-averaged F1-score of 0.73 indicates balanced performance across emotion categories, confirming that the proposed fusion framework maintains stable and meaningful discrimination under conventional evaluation settings despite the increased complexity of the CREMA-D dataset.

##### Performance under subject-exclusive 5-fold cross-validation

**Fig. 14:**
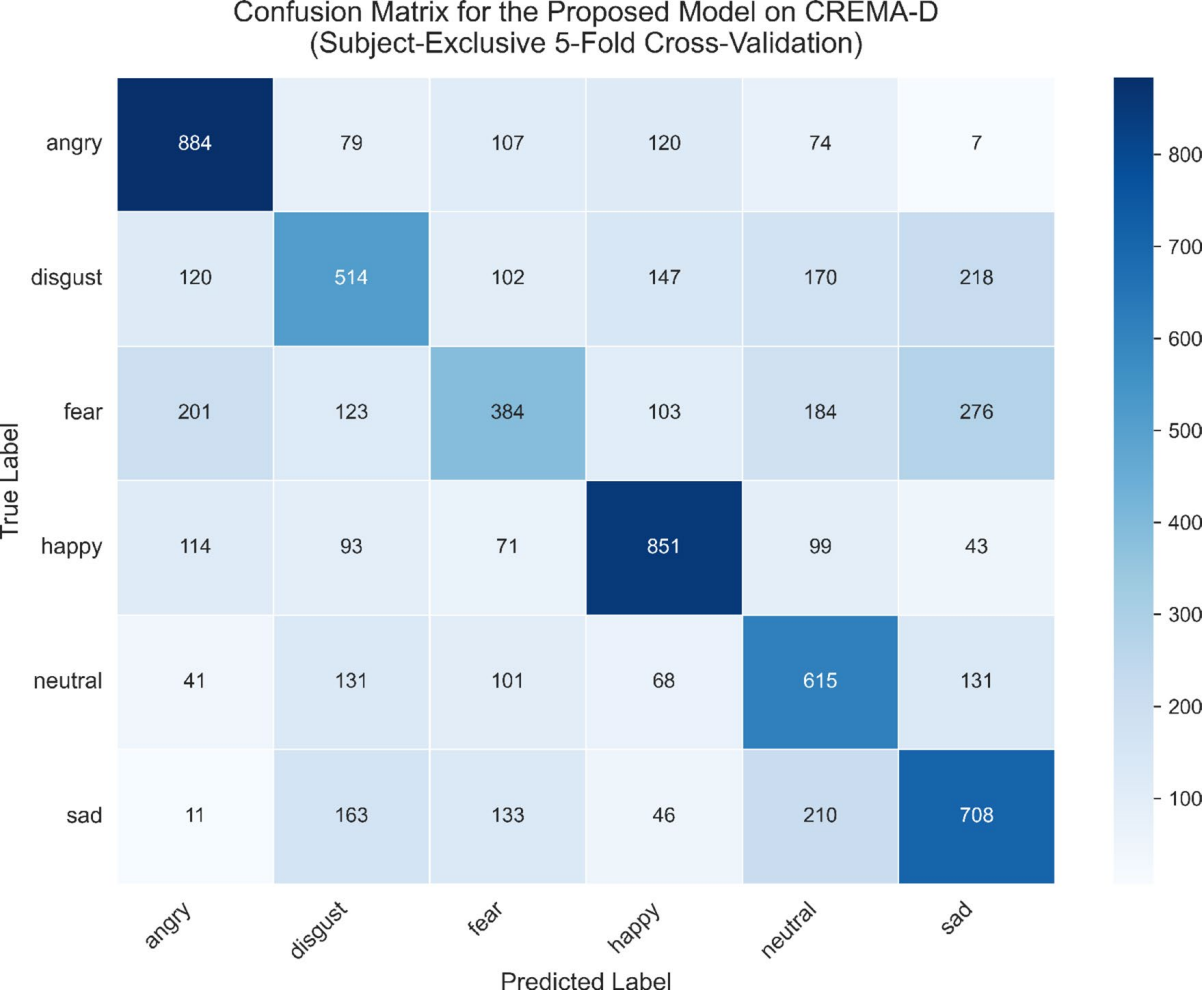
Confusion matrix of the proposed model on CREMA-D dataset (Subject-Exclusive 5-Fold Cross-Validation).

**Fig. 15:**
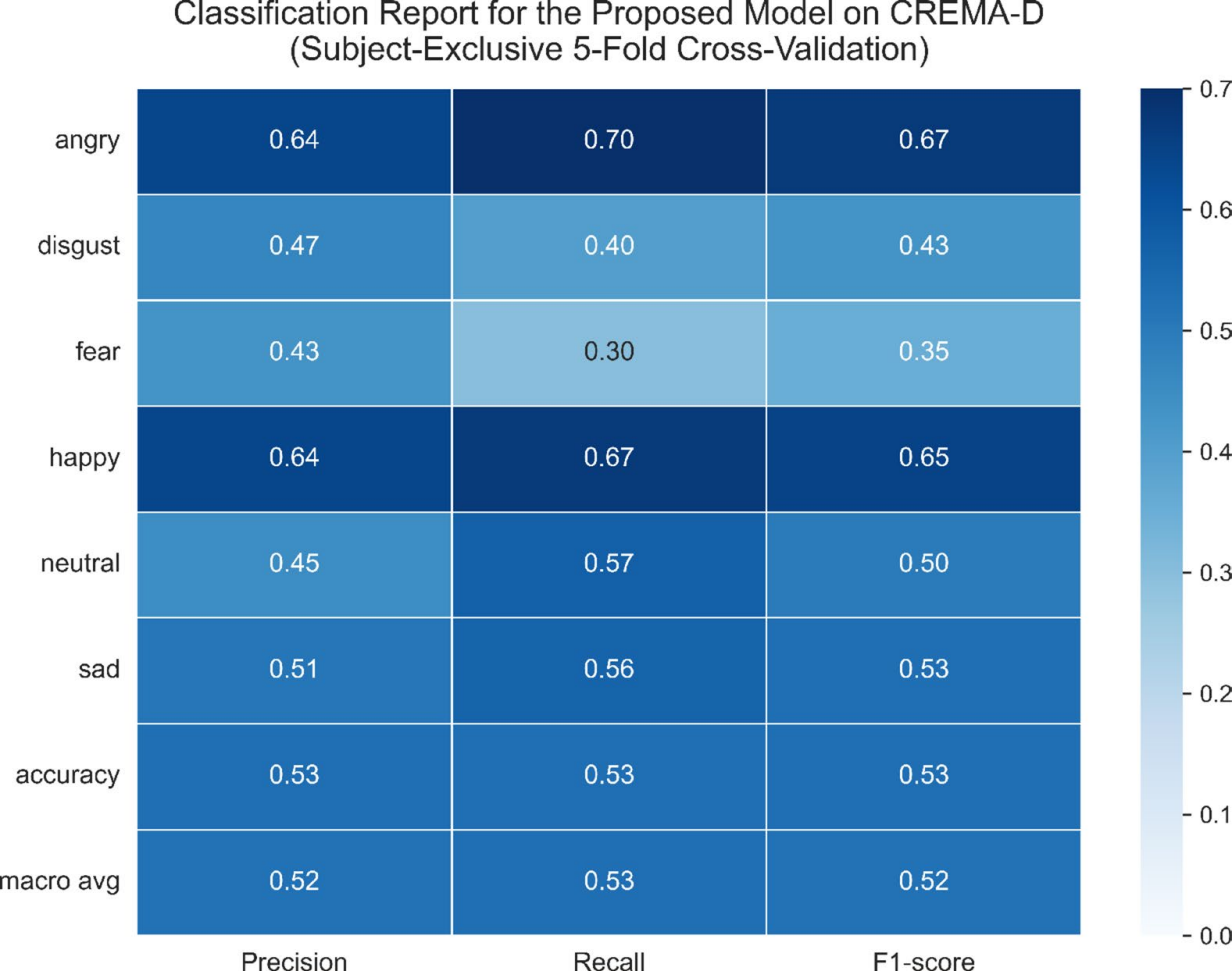
Classification report for the proposed model showing precision, recall, F1-score, and accuracy for each emotion class on the CREMA-D dataset (Subject-Exclusive 5-Fold Cross-Validation).

To further evaluate the generalization capability of the proposed framework under strict speaker-independent conditions, a subject-exclusive 5-fold cross-validation protocol was applied to the CREMA-D dataset, ensuring that samples from each speaker appear exclusively in either the training or testing folds. Under this evaluation setting, the proposed model achieved an average classification accuracy of 53.12% ± 2.65%, reflecting the increased difficulty of cross-speaker emotion recognition in a large-scale, speaker-diverse dataset. Figure 14 presents the confusion matrix obtained under the subject-exclusive 5-fold protocol, providing insight into the model’s class-wise behavior when evaluated on unseen speakers. Strong recognition performance is observed for highly expressive emotions such as Angry (884 correctly classified samples) and Happy (851 correctly classified samples), indicating relatively higher speaker-invariant consistency for high-arousal affective states. In contrast, lower recall values are observed for more subtle and acoustically overlapping emotions, particularly Fear and Disgust, which exhibit substantial confusion with neighboring affective categories. Notable misclassification patterns occur between Fear and Sad, Disgust and Sad, and Neutral and Sad, highlighting the intrinsic ambiguity of these emotional states in the CREMA-D dataset. Such confusions are expected given the wide variability in speaking styles, recording conditions, and emotional intensity across speakers, which amplify inter-speaker differences under subject-exclusive evaluation protocols. The classification report shown in Fig. 15 further corroborates these findings. The model achieves the highest F1-scores for Angry (0.67) and Happy (0.65), confirming robust recognition of high-energy emotions across speakers. Conversely, lower F1-scores are observed for Fear (0.35) and Disgust (0.43), reflecting their susceptibility to speaker-dependent acoustic variations. The neutral class attains a moderate F1-score of 0.50, consistent with its low expressive salience and overlap with adjacent emotional states. The macro-averaged F1-score of 0.52 indicates balanced but challenging performance under speaker-independent conditions, reinforcing the inherent difficulty of generalizing emotion recognition models across unseen speakers in the CREMA-D dataset.

##### Overfitting analysis & generalization

**Fig. 16:**
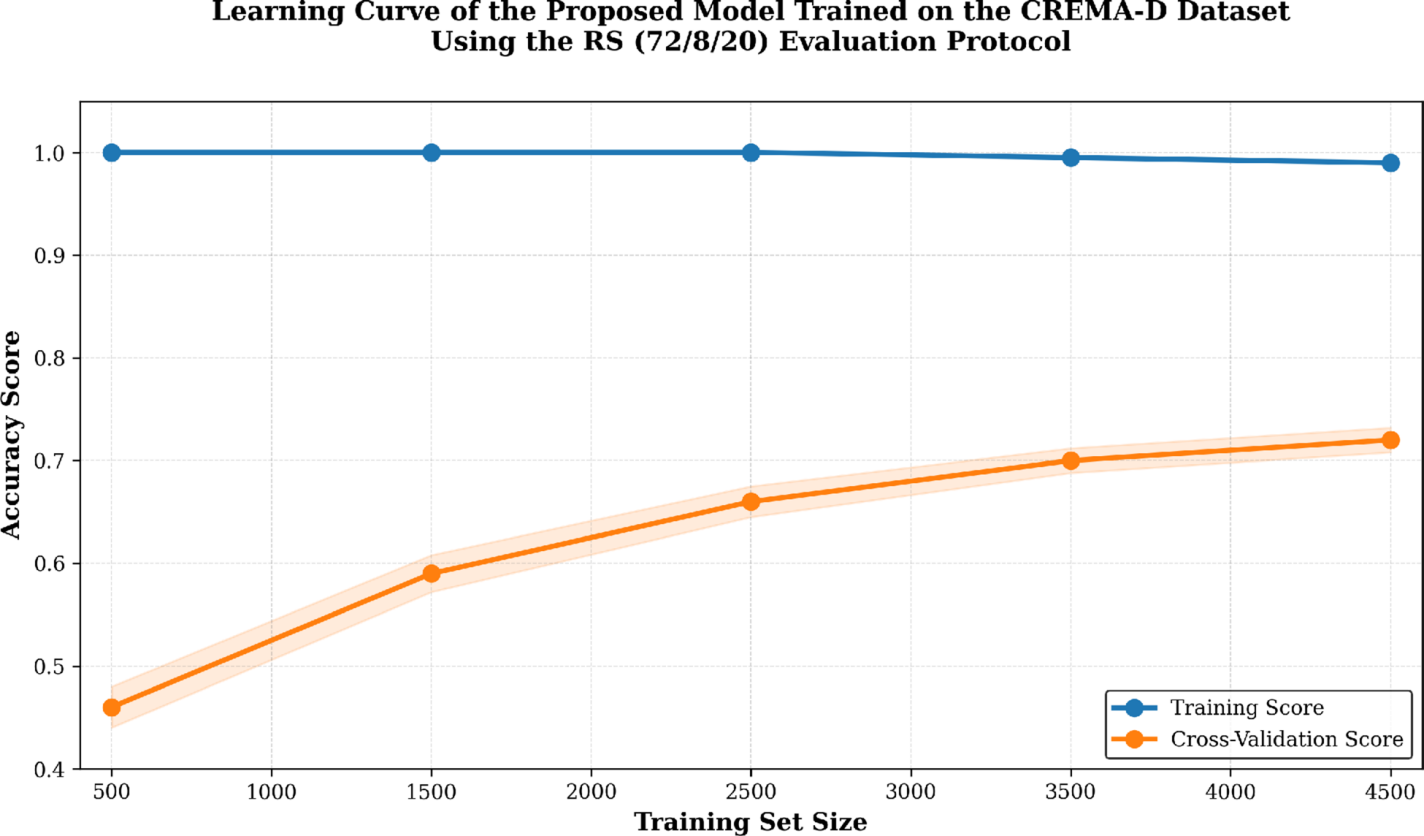
Learning curve of the proposed model trained on the CREMA-D dataset using the RS (72/8/20) evaluation protocol.

Figure 16 presents the learning curve of the proposed model trained on the CREMA-D dataset under the random split (RS 72/8/20) evaluation protocol. The training accuracy remains consistently high and close to saturation across all training set sizes, indicating that the model is able to effectively fit the training data under conditions where speaker overlap is present. In contrast, the cross-validation accuracy shows a steady increase as the number of training samples grows, rising from approximately 46% at smaller training sizes to over 72% when the full training set is utilized. This trend suggests that the model continues to benefit from additional data, with no clear indication of early saturation. A noticeable gap between training and validation performance is observed throughout the learning process. This gap can be partly attributed to the difference between fitting samples from overlapping speaker distributions and generalizing to held-out data within the same distribution. The gradual improvement of the validation curve, together with its relatively stable behavior, suggests that the model does not exhibit abrupt performance fluctuations as data size increases. As the training set expands, the validation performance moves closer to the training curve, indicating that increased data availability helps reduce the impact of data variability. This behavior appears to be consistent with the characteristics of the RS protocol, where speaker overlap simplifies the generalization task. At the same time, the persistence of a gap suggests that performance may still be influenced by dataset properties such as variability in expression and recording conditions, rather than being solely determined by model capacity.

**Fig. 17:**
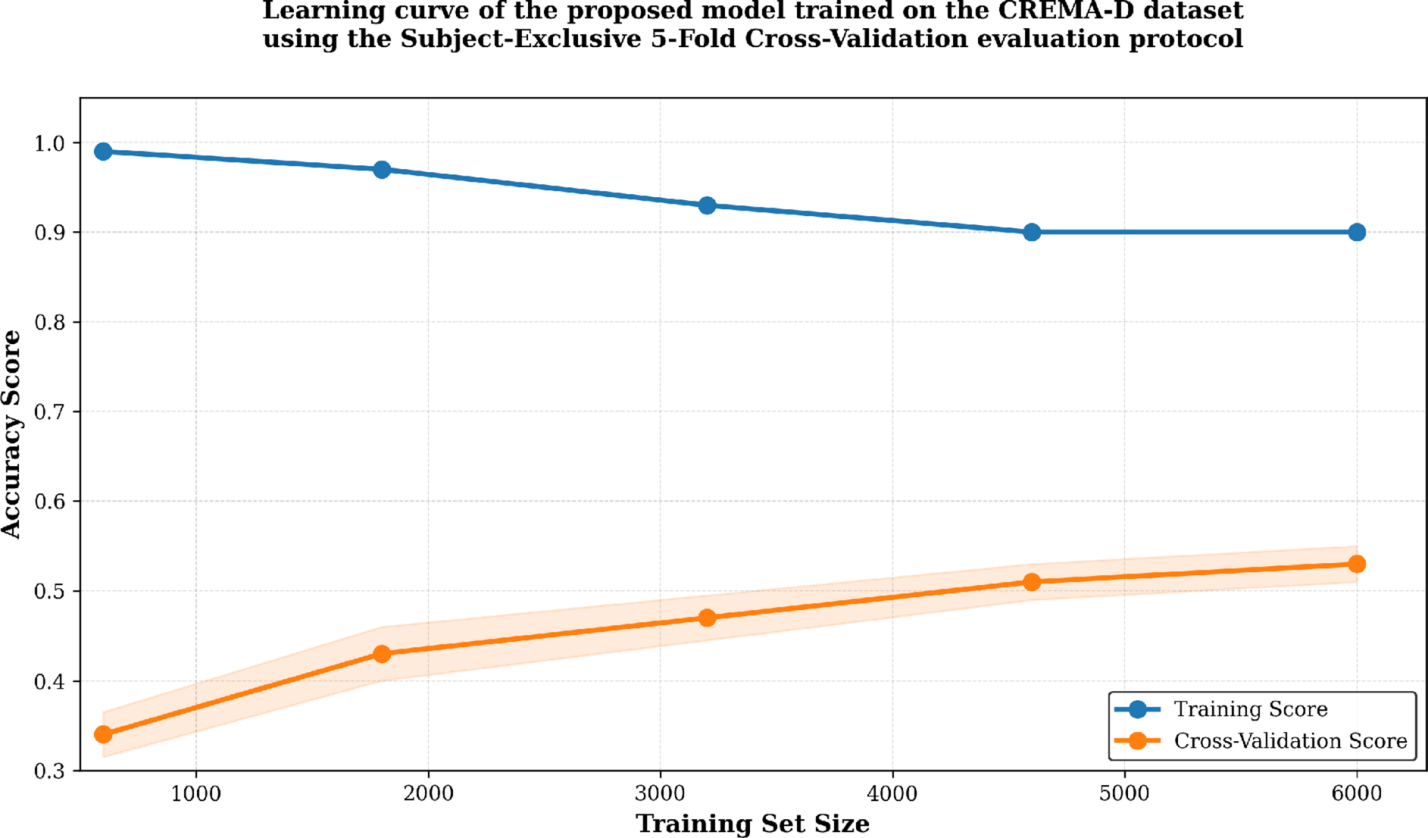
Learning curve of the proposed model trained on the CREMA-D dataset using the Subject-Exclusive 5-Fold Cross-Validation evaluation protocol.

Figure 17 illustrates the learning curve of the proposed model trained on the CREMA-D dataset under the subject-exclusive 5-fold cross-validation evaluation protocol. The training accuracy remains relatively high across all training set sizes, with a gradual decrease as the number of training samples increases. This behavior suggests that, as data diversity grows, the model becomes less dependent on fitting specific training samples and is exposed to a wider range of speaker variations. In contrast, the cross-validation accuracy on unseen speakers starts at a lower level and improves steadily with increasing training set size, reaching approximately 53% at full data utilization. This upward trend indicates that the model continues to benefit from additional speaker diversity, although the rate of improvement appears more gradual compared to random split settings. A persistent gap between training and validation performance is observed, which is expected under subject-exclusive evaluation. This gap can be attributed primarily to inter-speaker variability, where differences in vocal characteristics, facial expressions, and recording conditions introduce additional challenges for generalization. Rather than indicating instability, this behavior appears to reflect the increased difficulty of learning speaker-invariant representations from a limited and heterogeneous dataset. The steady improvement of the validation curve, together with the absence of abrupt performance fluctuations, suggests that the model maintains relatively stable learning behavior as more data are introduced. At the same time, the remaining gap indicates that performance is still influenced by dataset properties, such as variability in expression styles and recording conditions, and may benefit from further increases in data diversity rather than additional model complexity.

### Comprehensive performance comparison of model variants

Table 6 presents a comprehensive comparison of all unimodal and multimodal variants across two datasets under both conventional (Random Split) and speaker-independent evaluation protocols. Random-split results are reported only as an optimistic upper-bound reference and should not be interpreted as indicators of model generalization. All baseline methods were evaluated using the same preprocessing pipeline, feature normalization procedures, dimensionality reduction settings, and evaluation protocols to ensure a fair comparison. Under the Random Split (RS) setting, most fusion-based approaches achieve strong performance, particularly on RAVDESS, where visual-dominant and simple fusion strategies (e.g., concatenation and gating) benefit from speaker overlap between training and testing data. However, this trend does not consistently transfer to speaker-independent protocols, where a substantial performance drop is observed across all baseline variants, indicating limited robustness to inter-speaker variability. Unimodal models exhibit pronounced sensitivity to the evaluation protocol. While the visual-only model achieves high accuracy under RS, its performance deteriorates sharply under LOSO and subject-exclusive validation, highlighting the susceptibility of facial cues to speaker-specific appearance and expression patterns. Similarly, multiplicative fusion demonstrates unstable behavior. Among the baseline fusion strategies, concatenation and gated fusion exhibit relatively stronger stability under speaker-independent settings; nevertheless, both suffer noticeable degradation in accuracy and macro-F1, reflecting insufficient cross-modal coordination when speaker identity varies.

**Fig. 18:**
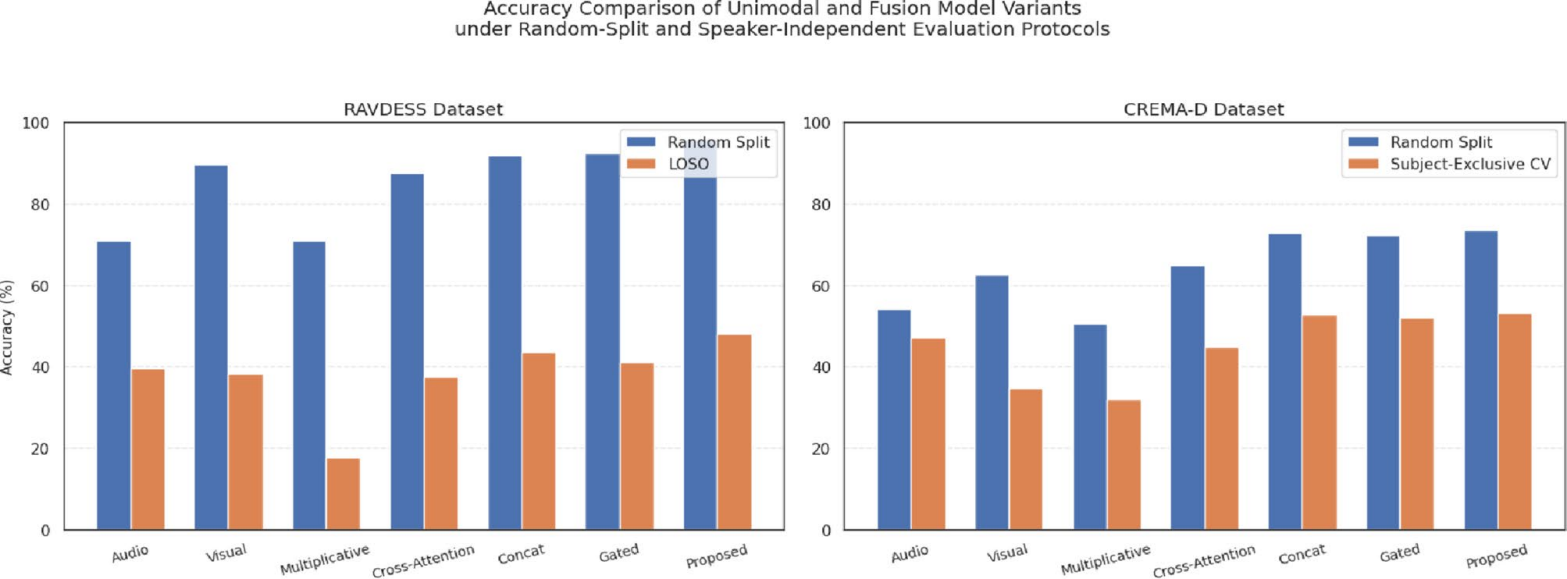
Accuracy comparison of unimodal and fusion model variants under random-split and speaker-independent evaluation protocol.

The proposed full model achieves higher accuracy and macro-F1 scores compared to all baseline variants across both datasets and evaluation protocols, as shown in Fig. 18. Notably, its advantage becomes more pronounced under speaker-independent evaluation, where it achieves the highest accuracy and macro-F1 on both RAVDESS (LOSO) and CREMA-D (subject-exclusive 5-fold CV). This improvement indicates that the proposed fusion design is more effective at learning speaker-agnostic multimodal representations, mitigating the sharp performance degradation observed in simpler fusion schemes. Unlike direct concatenation or isolated attention mechanisms, the proposed architecture maintains balanced contributions from audio and visual modalities while suppressing modality-specific noise. The observed performance improved through the complementary roles of the different fusion mechanisms. Simple concatenation provides a direct joint representation of audio and visual features – preserving the full feature space and enabling the model to exploit coarse cross-modal correlations^3^. In contrast, gated fusion introduces an adaptive, multiplicative weighting mechanism that dynamically modulates the contribution of each modality based on its reliability, which is especially useful when one modality is degraded or noisy^42^. Attention-based fusion further improves this by explicitly modeling cross-modal dependencies, allowing the model to focus on salient interactions between acoustic and visual cues rather than treating them independently^51^. Meanwhile, multiplicative fusion captures direct element-wise interactions between modalities, allowing the model to emphasize aligned feature activations that simple linear combinations cannot represent^52^. Collectively, these mechanisms address different aspects of multimodal integration – from basic feature aggregation to adaptive weighting and higher-order interaction modeling. The consistent performance gains observed for the full, multi-branch architecture suggest that these components contribute distinct yet complementary information, leading to a more expressive and balanced representation of emotional cues. This behavior also indicates that the improvement is not driven by any single fusion strategy in isolation, but rather emerges from the interaction between multiple fusion mechanisms, each capturing different aspects of cross-modal relationships. This integrated representation is particularly advantageous under speaker-independent conditions, where variability in expression style, facial dynamics, and vocal characteristics requires the model to draw on multiple coordinated modalities rather than a single dominant one. From a representation learning perspective, these fusion strategies operate at different levels of abstraction, providing complementary views of the input that may reduce reliance on any single interaction pattern and support more robust generalization.

Although the performance improvements under speaker-independent evaluation are more moderate compared to random-split settings, this behavior is expected given the increased difficulty of generalizing across unseen speakers. In such conditions, even modest gains are particularly meaningful, as they reflect improved robustness rather than overfitting to speaker-specific patterns. This indicates that the proposed architecture contributes to more stable and reliable performance under realistic evaluation scenarios, where achieving consistent improvements is inherently challenging.

These results are consistent with prior research emphasizing the limitations of conventional evaluation strategies in emotion recognition. In particular, several studies have shown that random split protocols may lead to overly optimistic performance estimates due to speaker overlap between training and testing sets. For example, Majkowski and Kołodziej^19^reported a substantial reduction in accuracy to 36.2% on the RAVDESS dataset under a subject-independent evaluation protocol, highlighting the difficulty of generalizing across unseen speakers. Similarly, Portal et al. and Chakhtouna et al^20,21^. emphasized the sensitivity of modern architectures to inter-speaker variability and demographic factors. In comparison, the proposed model achieves higher accuracy under speaker-independent evaluation (e.g., 48.06% on RAVDESS and 53.12% on CREMA-D), indicating improved robustness and a stronger ability to learn speaker-agnostic multimodal representations, while it is important to consider that both datasets employed in this study consist of acted emotional expressions recorded in controlled environments, and therefore the reported performance may not fully reflect the complexity, variability, and subtle dynamics of spontaneous emotions encountered in real-world conditions.

Overall, the results confirm that while several fusion strategies perform competitively under relaxed evaluation settings, robust generalization to unseen speakers remains a critical challenge. The proposed model addresses this limitation more effectively than existing baselines, validating its design choices and justifying its added architectural complexity.

**Table 6:** Comprehensive comparison of unimodal and multimodal models (Accuracy and Macro-F1) across RAVDESS and CREMA-D datasets under random split and speaker-independent evaluation protocols.

Model Variant	RAVDESS (RS)	RAVDESS (LOSO)	CREMA-D (RS)	CREMA-D (Subject-Excl. 5-Fold CV)
Audio-Only	70.83%/0.69	39.72% ± 10.99/0.38	54.06%/0.54	47.10% ± 3.39/0.46
Visual-Only	89.58%/0.90	38.19% ± 10.07/0.36	62.53%/0.62	34.70% ± 1.93/0.34
Multiplicative Fusion	70.83%/0.70	17.57% ± 5.83/0.15	50.64%/0.50	31.95% ± 1.89/0.30
Cross-Attention Fusion	87.50%/0.87	37.43% ± 9.45/0.35	64.88%/0.65	44.86% ± 1.32/0.44
Concatenation Fusion	92.01%/0.92	43.68% ± 10.66/0.39	72.67%/0.72	52.71% ± 2.53/0.52
Gated Fusion	92.36%/0.92	40.97% ± 11.15/0.35	72.20%/0.72	52.02% ± 2.28/0.51
Proposed Full Model	**95.83%/0.96**	**48.06% ± 9.76/0.45**	**73.54%/0.73**	**53.12% ± 2.65/0.52**

### Statistical validation under speaker-independent evaluation

To ensure that the performance gains achieved by the proposed multi-view fusion model are statistically reliable and not attributable to random variation, statistical validation was conducted exclusively under a speaker-independent evaluation protocol. All analyses in this section are based on Leave-One-Speaker-Out (LOSO) testing on the RAVDESS dataset, ensuring that all predictions are generated from speakers entirely unseen during training. To compare the proposed model against competing unimodal and fusion-based baselines, McNemar’s paired significance test was applied to the aggregated LOSO predictions. McNemar’s test is designed for paired nominal outcomes and evaluates whether two classifiers differ significantly in their error patterns when evaluated on the same set of samples. In this work, predictions are paired at the sample level and categorized as correct or incorrect relative to the ground-truth label, making McNemar’s test well-suited for speaker-independent evaluation.

For each pairwise comparison, McNemar’s test is computed using the contingency table defined by two quantities:

b, representing the number of samples correctly classified by the proposed model but misclassified by the baseline, and c, representing the number of samples misclassified by the proposed model but correctly classified by the baseline. The test statistic evaluates whether the imbalance between b and c deviates significantly from what would be expected under the null hypothesis of equal error distributions. For completeness, the exact formulation of the test statistic and the hypothesis definition are provided in Appendix A.

A statistically significant result indicates that the proposed model corrects a systematic, non-random subset of errors made by the competing approach. Because the proposed model was compared against six baseline variants, Bonferroni correction was applied to control the family-wise error rate, yielding an adjusted significance threshold of

$$\:{\alpha\:}_{corrected}=\frac{0.05}{6}=0.00833$$

#### McNemar test results

Table 7 summarizes the results of McNemar’s test for all model comparisons under the LOSO protocol. The proposed model demonstrates statistically significant improvements over the audio-only, visual-only, multiplicative fusion, cross-attention fusion, and gated fusion baselines, with p-values consistently below the corrected significance threshold. Notably, pronounced imbalances between b and c values (e.g., b = 495 vs. c = 84 for multiplicative fusion) indicate that the proposed model corrects a substantial number of systematic errors produced by these approaches. In contrast, no statistically significant difference is observed between the proposed model and the concatenation-based fusion approach after Bonferroni correction $\:p\:=\:3.78\:\times\:\:10^{-2}$. This outcome is expected, as concatenation already constitutes a strong fusion baseline, and its prediction patterns exhibit substantial overlap with those of the proposed system. Although the proposed model attains higher overall accuracy, the magnitude of the difference is insufficient to produce a statistically significant divergence in error patterns under the corrected threshold.

Overall, the statistical analysis indicates that performance gains are not uniformly significant across all comparisons. The absence of statistical significance in comparison with the concatenation-based approach highlights the strength of this baseline and suggests that improvements among advanced fusion strategies are relatively modest under speaker-independent evaluation. The relatively lower absolute accuracy values in this setting reflect the increased difficulty of generalizing across unseen speakers rather than a limitation of the model itself. In such scenarios, performance improvements are typically incremental, as models are required to capture speaker-independent patterns instead of relying on identity-specific cues. Consequently, moderate gains in accuracy and macro-F1 can be interpreted as indicators of improved robustness and more stable behavior across diverse speakers.

**Table 7:** McNemar test-based statistical significance analysis of the proposed multi-view fusion model compared to unimodal and fusion-based baselines on the RAVDESS dataset using speaker-independent Leave-One-Speaker-Out (LOSO) evaluation.

Model Variant	b	c	McNemar *p*-value	Significant (Bonferroni)
Audio-only	280	186	1.55 × 10⁻⁵	Yes
Visual-only	337	224	2.10 × 10⁻⁶	Yes
Multiplicative fusion	495	84	7.57 × 10⁻⁷²	Yes
Cross-attention fusion	303	173	2.70 × 10⁻⁹	Yes
Gated fusion	207	131	4.21 × 10⁻⁵	Yes
Concatenation fusion	169	132	3.78 × 10⁻²	No

### Regularization strategy and model complexity justification

Despite the relatively high architectural complexity of the proposed multi-view fusion framework, multiple design choices were explicitly adopted to mitigate overfitting and promote robust generalization. Rather than relying on a single regularization mechanism, overfitting control was enforced through a combination of evaluation protocols, architectural constraints, and data-driven validation strategies.

First, strict speaker-independent evaluation protocols were employed, including Leave-One-Speaker-Out (LOSO) on RAVDESS and subject-exclusive 5-fold cross-validation on CREMA-D. These protocols inherently act as strong regularization mechanisms, as they prevent any speaker overlap between training and testing sets and significantly reduce the likelihood of memorizing speaker-specific characteristics^19–21^. The consistent performance degradation observed when transitioning from random split to speaker-independent evaluation—without performance collapse—indicates controlled generalization^20^. Second, feature-level regularization was implicitly applied during the fusion process. High-dimensional visual representations extracted from facial frames were reduced using PCA, limiting redundant feature correlations and constraining the effective capacity of the visual branch^31,53^. On the classification side, ensemble learning via stacking was adopted, combining heterogeneous classifiers (SVM, Random Forest, and XGBoost). Such ensemble strategies are well known to reduce variance and improve generalization by aggregating complementary decision boundaries rather than relying on a single high-capacity classifier. Additionally, internal regularization was enforced within individual classifiers. The Random Forest model benefits from bootstrap aggregation, while the XGBoost classifier incorporates depth constraints, subsampling, and column sampling, all of which act as implicit regularizers. These design choices collectively restrict model complexity at the decision level, even when operating on high-dimensional fused representations. The learning curve analyses further support the effectiveness of these regularization strategies. Under the random split protocol, the validation accuracy increases monotonically with training set size and converges toward the training accuracy, exhibiting a bounded train–validation gap. Under speaker-independent protocols, while a larger generalization gap is observed—as expected—the test performance consistently improves with increased speaker diversity, indicating that the model continues to benefit from additional data rather than saturating due to memorization. This behavior suggests that the observed gaps are primarily driven by inter-speaker variability and dataset limitations, rather than excessive model capacity. Finally, the use of a large multi-view fusion design is justified by the intrinsic complexity of audio–visual emotion recognition. Emotional expressions are manifested through heterogeneous and complementary cues spanning acoustic prosody, facial dynamics, and cross-modal temporal interactions. The ablation and comparative analyses presented in Sect. 4.2 demonstrate that simpler variants (audio-only, visual-only, or single-fusion strategies) consistently underperform the full model. This confirms that the additional architectural components are not redundant but necessary to capture the multi-faceted nature of emotional expressions.

The proposed model architecture achieves a balanced trade-off between expressive power and generalization. The combination of strict evaluation protocols, ensemble-based regularization, dimensionality control, and empirical learning curve evidence demonstrates that the model’s complexity is both controlled and warranted by the task’s inherent difficulty.

### Limitations of the study

Despite the strong performance achieved by the proposed multi-view hybrid fusion model, several limitations should be acknowledged to properly contextualize the findings and outline directions for future work:

First, although both RAVDESS and CREMA-D were employed as primary datasets and evaluated using speaker-independent protocols, both corpora rely predominantly on acted emotional expressions performed by professional actors. While acted datasets facilitate controlled experimentation and clear emotional labeling, they do not fully capture the complexity of spontaneous emotional behavior observed in real-world scenarios. Acted emotions tend to be more exaggerated and acoustically or visually separable, whereas natural emotions are often subtle, ambiguous, and shaped by contextual factors such as fatigue, personality traits, social interaction, and situational dynamics. Consequently, even under speaker-independent evaluation, the reported performance may overestimate generalization to fully naturalistic environments^1,2,12,34^.

Second, despite improvements in speaker diversity achieved through the inclusion of CREMA-D, demographic and cultural coverage remains limited across both datasets. RAVDESS contains only 24 North American speakers with homogeneous linguistic and cultural characteristics, while CREMA-D—although larger and more diverse in age and gender—still reflects a constrained set of languages, accents, and cultural contexts. As a result, latent demographic and culture-specific biases may persist, limiting the robustness of the proposed model when applied to speakers from underrepresented linguistic or socio-cultural backgrounds. True cross-cultural and multilingual generalization, therefore, remains an open challenge^1,6,7,34^.

Third, the proposed multi-view architecture integrates multiple expressive fusion mechanisms, including multiplicative fusion and cross-attention, which substantially increase model capacity. While the multi-view design improves stability by aggregating complementary representations, dataset scale remains a critical limiting factor. Under speaker-independent evaluation on both RAVDESS and CREMA-D, some fusion branches exhibit unstable or inconsistent behavior, reflecting the data-hungry nature of high-dimensional interaction models. This limitation highlights the need for training on larger-scale, less constrained multimodal corpora to fully exploit the representational power of complex fusion strategies^5,52,53^.

Finally, although the stacking ensemble classifier improves overall performance and stability, the computational complexity of the full system is higher than that of single-branch models. This is primarily due to the cost of deep visual feature extraction and the parallel evaluation of multiple fusion branches. Real-time deployment may require further optimization, model compression, or pruning techniques to reduce inference time without compromising accuracy^47,54^.

Overall, these limitations highlight the necessity of future research focused on spontaneous, large-scale, and cross-cultural multimodal datasets, as well as the exploration of domain adaptation, cross-dataset generalization, and computational efficiency strategies to enhance the real-world applicability of advanced multimodal emotion recognition systems.

## Future work

Our multi-layer model performs efficiently in emotion recognition, but there are several directions in which we can improve its capabilities.

- Expanding the dataset on which the model was trained will make our model more general to include a larger number of speakers and dialects, which ensures better generalization and higher model performance. Similar to the findings of Altaibek et al^55^. and Wu et al^2^., expanding the training dataset to include more speakers, dialects, and emotional contexts can improve generalization and robustness across diverse populations.
- Integrating the model with temporal modeling through architectures such as Bi-LSTM or Transformer-based networks^47^ can help capture sequential emotional patterns across video frames more effectively. As demonstrated by Jin and Zai^9^, and Song and Zhou^15^, employing temporal models such as Bi-LSTM or Transformer architectures enables the system to capture sequential emotional patterns across video frames more effectively.
- Experimenting with real-time emotion recognition systems that operate efficiently on edge devices, enabling practical deployment in human–computer interaction environments. Following the approach of Kotkar^56^and Mocanu et al^5^., developing real-time multimodal systems optimized for edge devices can enhance practical deployment in human–computer interaction environments.
- The model performance will improve when we add more input modalities to the system because text and physiological signals provide useful information beyond audio and visual data. Previous studies have shown that integrating additional modalities such as text or physiological signals can provide complementary emotional cues, leading to more accurate emotion recognition^7,38,57^.
- The model’s performance will improve through domain adaptation and self-supervised learning techniques, which enable fine-tuning of the proposed model under noisy conditions. Recent studies, such as Siriwardhana et al^47^. and He et al^54^.. Highlighted that domain adaptation and self-supervised learning enable models to generalize better under noisy and unseen conditions.

## Conclusion

This study presented a robust multimodal emotion recognition framework that integrates audio and visual information through a novel multi-view hybrid fusion architecture. The proposed system combines handcrafted acoustic features with deep visual representations extracted using a VGGFace–ResNet50 backbone and jointly exploits four complementary fusion mechanisms—concatenation, gated fusion, cross-modal attention, and interactive multiplicative fusion—within a unified stacking-based ensemble classifier. Experimental evaluations were conducted under both random data splitting and strict speaker-independent protocols, providing complementary perspectives on model behavior. While random split experiments demonstrate the upper-bound performance of the proposed architecture under controlled conditions, speaker-independent evaluation—implemented via Leave-One-Speaker-Out (LOSO) on RAVDESS and subject-exclusive validation on CREMA-D—serves as the primary and more realistic assessment of generalization to unseen speakers. Under these challenging conditions, the proposed multi-view model consistently outperformed unimodal baselines and most fusion variants, achieving the highest speaker-independent accuracy on RAVDESS and demonstrating strong generalization behavior across both datasets. A comprehensive ablation analysis confirmed that integrating multiple fusion strategies within a single multi-view architecture yields more robust and discriminative emotional representations than relying on individual modalities or single fusion mechanisms. Furthermore, statistical validation using McNemar’s paired significance test under LOSO evaluation verified that the proposed model achieves statistically significant improvements over audio-only, video-only, multiplicative fusion, cross-attention, and gated fusion baselines, while exhibiting comparable performance to strong concatenation-based fusion. These findings demonstrate that the observed gains are not merely numerical but reflect meaningful and systematic error reduction under speaker-independent testing. Despite these advances, the study also highlights important challenges inherent to multimodal emotion recognition. Both RAVDESS and CREMA-D are predominantly composed of acted emotional expressions, which may limit direct transfer to spontaneous real-world scenarios. Additionally, high-capacity fusion mechanisms remain sensitive to dataset scale, and speaker-independent evaluation exposes substantial inter-speaker variability that remains difficult to model fully.

This work provides strong empirical evidence that combining diverse fusion paradigms within a unified multi-view framework leads to more reliable, noise-resilient, and generalizable emotion recognition. By explicitly contrasting random split and speaker-independent evaluation protocols, the study underscores the importance of realistic assessment strategies for advancing multimodal emotion recognition toward real-world deployment.

## Data Availability

The datasets analyzed during the current study are publicly available: The RAVDESS dataset is available through the Zenodo repository at: https://zenodo.org/records/1188976. The CREMA-D is publicly available and can be accessed from its official repository at: https://github.com/CheyneyComputerScience/CREMA-D.

## Code availability

The resources associated with this study are publicly available at: https://github.com/MohameddEzzatt/multimodal-emotion-recognition-reproducibility and via Zenodo at: 10.5281/zenodo.20705879. The repository includes extracted multimodal feature representations, predefined speaker-independent LOSO train/test splits, trained models, preprocessing details, library versions, and implementation resources related to the experiments presented in this work.
